# Geographical Pattern Evolution of Health Resources in China: Spatio-Temporal Dynamics and Spatial Mismatch

**DOI:** 10.3390/tropicalmed7100292

**Published:** 2022-10-10

**Authors:** Yong Zhou, Kaixu Zhao, Junling Han, Sidong Zhao, Jingyuan Cao

**Affiliations:** 1 Graduate Institute of National Development, National Taiwan University, Taipei 10617, Taiwan, China; 2 College of Urban and Environmental Science, Northwest University, Xi’an 710127, China; 3 Clinical Laboratory, The Affiliated Taizhou People’s Hospital of Nanjing Medical University, Taizhou 210029, China; 4 School of Civil Engineering and Architecture, Guangxi University, Nanning 530004, China; 5 School of Architecture, Southeast University, Nanjing 210096, China; 6 School of Biological Science and Medical Engineering, Southeast University, Nanjing 210096, China

**Keywords:** medical resource, geospatial health, spatial analysis, driving mechanism, China

## Abstract

(1) Background: The rational allocation of limited medical resources is the premise of safeguarding the public health. Especially since the outbreak of COVID-19, the evolution dynamics and spatial mismatch of medical resources have been a focal and frontier issue in academic discussions. (2) Methods: Based on the competitive state model and spatial mismatch index, this paper uses GIS and Geodetector spatial analysis methods and three typical indicators of hospitals, doctors, and beds to conduct an empirical study on the evolutionary characteristics and degree of mismatch in the geographic pattern of health resources in China from 2010 to 2020 (the data are from official publications issued by the National Bureau of statistics in China), in two dimensions of resource supply (economic carrying capacity) and demand (potential demand or need of residents). (3) Results: The spatial pattern of health resources at the provincial level in China has been firmly established for a long time, and the children and elderly population, health care government investment, and service industry added value are the key factors influencing the geographical distribution of health resources. The interaction between the different influence factors is dominated by bifactor enhancement, and about 30–40% of the factor pairs are in a nonlinear enhancement relationship. Hospital, doctor, and bed evolution trends and the magnitude and speed of their changes vary widely in spatial differentiation, but all are characterized by a high level of geographic agglomeration, heterogeneity, and gradient. Dynamic matching is the mainstream of development, while the geographical distribution of negative and positive mismatch shows strong spatial agglomeration and weak spatial autocorrelation. The cold and hot spots with evolution trend and space mismatch are highly clustered, shaping a center-periphery or gradient-varying spatial structure. (4) Conclusions: Despite the variability in the results of the analyses by different dimensions and indicators, the mismatch of health resources in China should not be ignored. According to the mismatch types and change trend, and following the geographic differentiation and spatial agglomeration patterns, this paper constructs a policy design framework of “regionalized governance-classified management”, in line with the concept of spatial adaptation and spatial justice, in order to provide a decision making basis for the government to optimize the allocation of health resources and carry out health spatial planning.

## 1. Introduction

Public health and well-being have been a frontier issue of academic research and the focus of social service work of government departments. Especially in the context of the COVID-19 outbreak [1,2], which has brought a great impact on people’s lives and social economy all over the world, how to rationally allocate the limited health resources (Medical Resources) has drawn wide attention from the public and scholars [3,4]. The United Nations ranks “good health and well-being” third among its 17 SDGs (Sustainable Development Goals) and proposes the goal of “ensuring universal access to well-being and health care and achieving universal health coverage” (SDG3) [5]. Health resources are scarce, basic, and public social services, and improving the spatial balance and equity of health resources is a vital target of governments and international organizations [6,7]. As the quality of life improves the population’s demand for health services, which is on the rise, leading to the fact that the capacity to supply health resources in many countries, especially in developing countries, it is facing great challenges against the background of increasingly high medical costs [8,9]. At present, the shortage of health resources goes hand-in-hand with their waste across the world, which, in essence, reflects the imbalance between the demand and supply of health resources [10]. Therefore, this paper analyzes the dynamic changes, geographic distribution characteristics, and degree of spatial mismatch of health resources in both supply and demand dimensions and proposes coping strategies to solve the mismatch, thus making it highly valuable for academic study, while providing a decision making basis for the government to encourage accurate investment and the rational allocation of health resources [11].

Health resources are an important part of public services, and their balanced spatial distribution matters for the residents’ access and fairness to medical services, as well as the sustainable economic development of the region and even the country. Since the reform and opening up, the capacity of medical services in China has been greatly improved [12], but the problems of uneven supply and demand of health resources and their spatial inequality are still very prominent, especially the unreasonable allocation between regions and urban and rural areas [13]. To this end, the State Council of China has proposed in the *“Health China 2030” Planning Outline* to integrate health into all policies and safeguard and protect people’s health in an all-round and full-cycle way. The central government’s implementation of the “Healthy China” strategy requires local governments to optimize the allocation of health resources (to better balance the supply of resources and demand for services by adjusting the quantity, structure, and geographical distribution pattern of health resources to improve the efficiency of resource utilization and health protection capacity). The rationality of geographic distribution and spatial allocation of health resources has a great influence on the ability to protect and improve the health of the population. Therefore, it is of great significance to analyze the geographical pattern, spatio-temporal evolution dynamics, mismatch characteristics of health resources, and coping strategies for the formulating scientific health care policies, in order to plan and build a “Healthy China” [14].

As one of the world’s largest developing countries, China should comply with the basic demand-oriented principle emphasized by the World Health Organization (WHO) in the allocation of health resources and construction of its service system, while taking the actual carrying capacity of economic development on resource supply into account [15,16]. This paper focuses on the following questions: (1) What are the regular characteristics of the spatio-temporal evolution dynamics of inter-provincial health resources in China? (2) How to quantitatively evaluate the degree and type of spatial mismatch of health resources in China in both the supply (economic carrying capacity) and demand (population potential consumption) dimensions? (3) What are the main factors influencing the geographical distribution of health resources and their interactive relationships? (4) How to design the spatial zoning policy for health resources?

To address the above issues, the follow-up of this paper consists of five parts. The first part is Literature Review, which systematically analyzes the key contributions and limitations of available papers and proposes the starting point of this study. The second part is Materials and Methods, which defines the study area in this paper, explains the selection of the research methods and variables and their data sources, and designs the research steps and ideas. The third part is Results, which quantitatively analyzes (using Boston consulting group matrix [17] and spatial economic model [18]) the dynamics and patterns of spatio-temporal evolution of health resources at the provincial level in China, analyzes the characteristics of spatial mismatch in both dimensions of supply and demand, and reveals the forces of influence factors and their interactive relationships, based on Geodetector, to provide a basis for policy design. The fourth part is the Discussion, which presents a comparative analysis of the main viewpoints of this paper against available papers (external evidence) and proposes a spatial strategy for the sustainable development of health resources. The fifth part is the Conclusions, which summarize the key views, innovations, and limitations of this paper and looks ahead to the international value of this study and the future work.

## 2. Literature Review

### 2.1. Medical Resource Change and Allocation Spatial Inequality Analysis Are the Mainstream, and the Research on Geospatial Health Is Insufficient

There are a growing number of studies on geographic inequality, spatial accessibility, allocation efficiency, spatio-temporal evolution characteristics of medical resources, and their influence factors, mostly using methods such as the Gini index, Lorenz curve, Theil index, and DEA. For example, Shaltynov [19] evaluated the spatial inequality and accessibility of primary health resources in Kazakhstan using the Gini index and Lorenz curve. Behr [20] analyzed the quantitative evolution characteristics of health resources in France and made a comparison and correlation analysis with demographic and epidemiological parameters. Omrani-Khoo [21] analyzed the degree of inequality and inequity in the distribution of health resources using the Gini coefficient, coupled with concentration and Robin Hood indices. Dong [22] analyzed the characteristics of regional distribution and inequality in health-resource allocation at hospitals and primary health centers in Shanghai using the Theil index. Chai [23] argued that regional disparities and imbalances in health resources in China are highly characteristic and have a significant impact on mortality. Fang [24] analyzed the impact of health care system reform on county-level health resource allocation and service utilization in China, based on the data from 1110 hospitals. Yang [25], Retzlaff-Roberts [26], and Liu [27] evaluated the efficiency of health resource allocation by data envelopment analysis. Giraldes [28] analyzed the allocation efficiency of medical resources in Portugal by marginal met need approach.

GeoHealth is currently emerging as a research paradigm [29], providing information, data, and rationale for health planning and policy design [30]. Brazil has established the Primary Care Information System to collect basic healthcare information from different regions, in an attempt to provide a georeferenced system for decision making by authorities [31]. Maire [32] stated that the health resources allocation model integrates the mechanisms by which geographic and demographic characteristics influence health resources. Anselmi [33] evaluated the horizontal and vertical equity of the geographical distribution of health expenditure (HE) between regions by benefit incidence analysis. McIntyre [34] proposed a broad-based area deprivation index and analyzed its geographical pattern and its impact on the allocation of health resources in South Africa. Hall [35] evaluated the performance of medical resource allocation in Canada and its match with demand using a cross-sectional approach and the Resource Allocation Performance Assessment Tool. However, overall, the influence of geographers and planners in the health resource policy arena is still limited, and there are still many barriers and blind spots in how to integrate the results of geospatial health analysis into the health planning and policy design process. Medical resources are special consumer and public articles, and their input and allocation should meet the needs of residents and bearing capacity of regional economic development. However, the available papers generally focus on the study of the “simplicity” of the geographical allocation of medical resources and its influence factors, without correlating their geographical distribution with population demand and economic carrying capacity, resulting in weak guidance for health planning, and policy design practice.

### 2.2. Qualitative, Statistical and Regression Analysis Are the Mainstream, and Lack of Support for Evidence-Based Decision Making

Most of the studies are conducted using qualitative and empirical analysis methods represented by questionnaires, conference interviews, case studies, and direct observation, and there is a growing number of papers on regression model analysis and cost-benefit comparative analysis based on statistics [36,37]. For example, Giacomini [38] proposed a qualitative investigation approach to the fairness of health resource allocation (fairness), based on the grounded theory. Kolasa [39] discussed the impact of different population preferences on the allocation of health resources through case studies. Frew [40] analyzed what and how economics evidence and methods are applied in the field of health resource allocation in the UK, based on in-depth interviews and observational methods. Freedman [41] analyzed the impact of state grant funding on the sustainability of health resource provision using semi-structured key informant interviews methods. Ahlert [42] and Furnham [43] analyzed the impact of moral intuitions and ethical ideology on health resource allocation using an experimental questionnaire method. Ma [44] analyzed the inequality of medical resource allocation in township health centers in China using questionnaires and direct observation methods. Bhattacharyya [45] analyzed the district-based health resource allocation decision making process in India, based on direct observations of key decision-making meetings and qualitative interviews with key informants). Krones [46] analyzed the attitudes of German inpatients, regarding the fairness of health resource allocation, by means of a structured questionnaire with patients. Smith [47] analyzed decision makers’ perceptions on the process of allocating health resources in Canada by online survey. Pourat [48] evaluated the differences in the quality of health resources between urban and rural areas in the United States, based on generalized linear regression models. Avelino [49] analyzed the level of health resource management in different Brazilian cities using a multiple regression model. Jain [50] analyzed the impact of climate change on health resource planning in India using multiple linear regression model and constructed a framework for evidence-based decision making of resource allocation on the basis of climate parameters. Lopes [51] analyzed the association between the uneven distribution of health resources and economic-social factors (the resident population, social vulnerability index, and municipal human development index) in Brazil using descriptive and statistical methods. Owili [52] and Sun [53] analyzed the impact of professionals and health care system reform on the allocation of health resources in Kenya and China using a structural equation model. Pinho [54] and John [55] critically analyzed the cost-effectiveness analysis (CEA) approach and further proposed novel solutions that do not discriminate against people with disabilities.

Reliable data and effective methods are essential for health planning and government decision making, especially in developing countries where health resources are scarce [56]. The introduction and innovation of research methods have been of interest to scholars. For example, Rivero-Garcia [57] proposed a secure approach to monitoring emergency health resources, and Pichon-Riviere [58] analyzed the link between health technology assessment (HTA) and resource allocation decisions in Latin America using design-thinking methodology. However, as Bekemeier [59] and Gong [60] argued, providing valid data and evidence for government health resource allocation decisions remains difficult, and decision makers in the health planning practice tend to rely on empirical or qualitative decision models, instead of evidence-based decision making, thus reducing the rationality and credibility of decisions [61]. The applicability and usefulness of qualitative research and regression statistical analysis are facing increasing challenges in the process of evidence-based decision making for regional allocation of health resources. For example, in the current era of big data, qualitative and qualitative research is increasingly weakened in guiding policy design, while quantitative analysis is showing its greater value. However, differences in the modeling paradigms or parameter designs adopted by the models used in different papers have led to a great variation in the analytical results of empirical studies with different methodologies and cases, as well as increasing difficulties in the application of the findings. For government officials, for example, the dynamics and spatio-temporal evolution characteristics of health resource allocation are the basis for developing management policies, and the three are interrelated; however, scholars tend to place their focus on one of the three, in general, and the separation of the three results in a “disconnect” between theoretical research and practical needs. Therefore, it calls for the introduction or creation of a new technical framework that integrates the three parts of “spatio-temporal dynamics-mismatch state-policy design” in the health resource allocation, accompanied by use of analysis results and conclusions as the basis for the allocation and planning of health resources by the government [62].

## 3. Materials and Methods

### 3.1. Study Area

**Figure 1:**
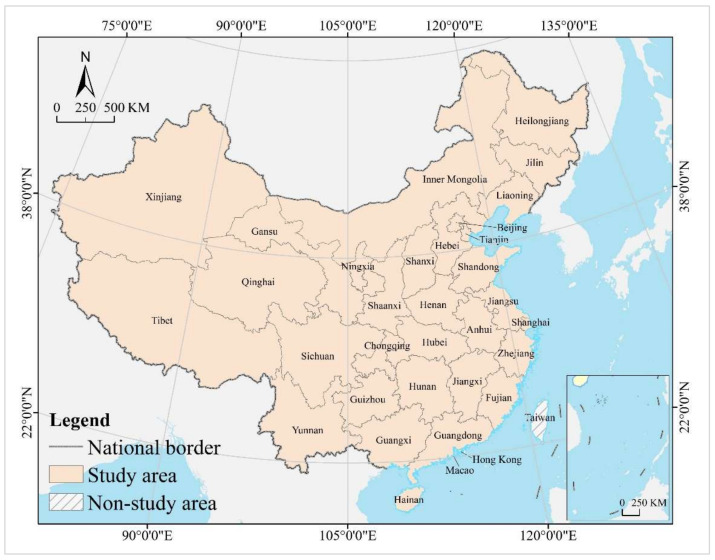
Study area.

The study area is mainland China, with a geographical coverage of 31 provincial-level administrative regions, including 22 provinces, 5 autonomous regions, and 4 municipalities directly under the central government. It does not include Hong Kong and Macao special administrative regions and Taiwan province, as their statistical caliber of data differs somewhat from that of mainland China (Figure 1).

Hong Kong, Macao, and Taiwan regions of China have a great deal of autonomy, especially Hong Kong and Macao, which have implemented the principle of “one country, two systems” (a basic state policy proposed by the Chinese government to achieve peaceful national reunification, meaning that, under the premise of one China, the main body of the country insists on the socialist system, while Hong Kong, Macao, and Taiwan maintain the original capitalist system for a long time). The difference in social systems has led to the fact that the scope, subjects, and caliber of statistics in Hong Kong, Macao, and Taiwan are quite different from those in mainland China. In 2010, China had 936,900 hospitals, 8,207,500 doctors, and 4,768,000 beds, which increased to 1,022,900, 13,475,000, and 9,107,000, respectively, by 2020, with an average annual growth of 0.88, 5.08, and 6.64%.

### 3.2. Research Steps and Data Sources

**Figure 2:**
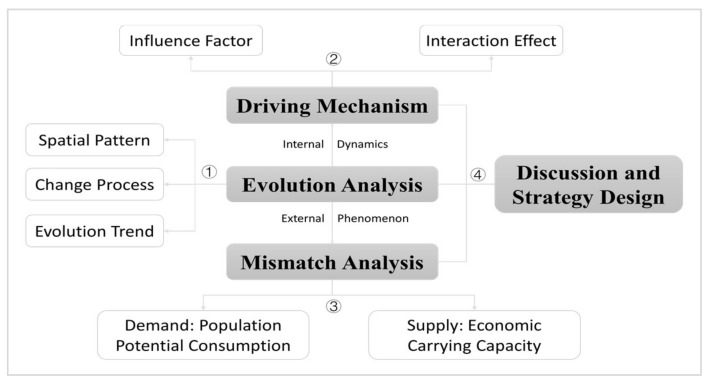
Research steps.

The first step is to analyze the spatio-temporal evolution characteristics of health resources. Using GIS tools and Boston Consulting Group matrix, we try to reveal the process of change and spatial pattern of medical resources at the provincial level in China. Health care institutions, doctors and health personnel, and beds in health care institutions reflect the level of health resource development in dimensions of organization, size, and capacity, respectively, and play a fundamental role in the health care service system [63,64]. Health care institutions include hospitals, health care institutions at grassroot level, and specialized public health institutions, which are referred to as “Hospitals” in this paper. Human resources include health technical personnel, village doctors and assistants (other), administrative staffs, and logistics technical workers, which are referred to as “Doctors”. Beds in health care institutions are referred to as “Beds” (Figure 2).

The second step is to analyze the match of health resources with population growth and economic development using a spatial mismatch model. Health resources are a guarantee of individual health and well-being, and also directly affect sustainable economic development, so changes in health resources should match the needs of the population and carrying capacity of the economy [65,66]. As health resources are scarce and limited, how to maintain high-quality health services is a huge challenge for the government, even for developed countries in Europe and the United States. Health is the most basic prerequisite for human survival and growth; for many countries, especially developing countries, there is a large contradiction between the supply of health resources and demand for services. Therefore, a normative technical framework is needed, regarding the spatial allocation of health resources [67], to guarantee a reasonable and balanced access to health care services for the population of all (or at least most) regions. Besides, China is currently in the transition phase from high economic growth to high-quality development (In other words, the goal orientation of economic development has changed from scale and speed to green, ecological, innovative, shared and competitive). Economic development is a prerequisite and guarantee for promoting health and achieving national health goals; in turn, reasonable growth in medical investment will also stimulate economic growth. Excessive investment in health resources may be a burden on economic development, but insufficient investment may lead to social discontent and threaten sustainable regional development. Therefore, measuring the matching relationship or mismatch degree of the medical resource changes with population growth and economic development allows for rational allocation and utilization of limited health resources, thus facilitating a dynamic balance between supply and demand, as well as between equity and efficiency, to encourage synergistic development of medical resources with population and economy [68,69]. Population and GDP are commonly used indices to measure regional population size and economic development level, and they are used in this paper to characterize the demand potential and supply capacity of health resources.

The third step is to analyze the factors affecting the geographical distribution and spatial pattern of medical resources using the Geodetector method. The geographical distribution of medical resources is influenced by a combination of economic strength, social environment, and health investment, including size of the economy, development stage, industrial structure, urbanization, population structure and level of education, government and resident medical investment, and social medical funds [70,71,72]. This paper uses service industry added value, per capita GDP, government revenue, social consumption to characterize economic factors urbanization rate, children and elderly population, high quality (university or above) population to characterize social factors, and it uses health care government investment, residents’ medical services consumption, and medical insurance fund expenditure to characterize investment factors (Table 1).

The fourth step is to propose an optimal strategy for health resource allocation, based on the analysis results, to provide guidance for policy design. The data used in this paper mainly comes from the *China Statistical Yearbook* published by the China’s National Bureau of Statistics (http://www.stats.gov.cn/tjsj/ndsj/ (accessed on 12 May 2022)) and *China Health and Wellness Statistical Yearbook* by the National Health Commission (https://data.cnki.net/Yearbook/Single/N2022010155 (accessed on 27 May 2022), with some missing data from the statistical yearbooks and bulletins of provincial administrative regions (Appendix A Table A1 and Table A2 is the sum standardized data). *China statistical yearbook* and *China health and wellness statistical yearbook* are the most authoritative and reliable statistical publications issued by the Chinese government. The former contains comprehensive statistics on the economic, social, ecological, and health services (including medical and health institutions, health officers and facilities, disease control, and health costs) of all provinces, autonomous regions, and municipalities directly under the central government in China, and the latter provides professional statistics that reflect the level of development of China’s medical and health services and health status of the population.

### 3.3. Research Methods

#### 3.3.1. Boston Consulting Group Matrix

**Figure 3:**
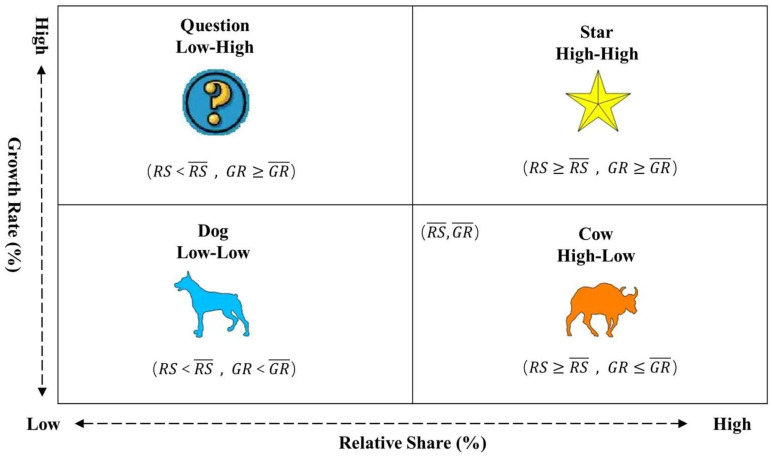
Boston Consulting Group matrix and decoupling index.

Boston Consulting Group matrix, created by Bruce Henderson, is the classic analytical approach in business management. For companies that provide more than one type of products or services, their sustainability should be evaluated in an integrated way through portfolio analysis among their businesses, since each business has different market positions and value advantages. In the Boston consulting group matrix-based analysis, the horizontal coordinate represents the relative market share of the company’s revenue, and the vertical coordinate represents the average annual growth rate of the revenue, with the average or a set value (e.g., 0.5 or 10%) as the threshold to classify the company’s business into four types, i.e., star, cow, question, and dog. Boston Consulting Group matrix is used in this paper to analyze China’s provincial health resources, with the aim of precisely analyzing their spatio-temporal evolution characteristics. By calculating the relative share (RS) and average annual growth (GR) of medical resources of the provinces in China by Equations (1) and (2), we classify the 31 provinces into four types of star, cow, question, and dog, with their average value ($\bar{RS}$ and $\bar{RS}$) as the threshold (Figure 3) [73].

(1) $$RS=\frac{X_{i}}{X_{i-max}}\times 100\%$$

(2) $$GR=\left(\sqrt[{t}]{\frac{X_{i-end}}{X_{i-base}}}-1\right)\times 100\%$$

where RS and GR represent the relative share and growth rate, respectively, used to reflect the relative position and degree of change of medical resources of a province in China; $X_{i}$ represents the medical resources of province i, $X_{i-max}$ represents the maximum value of medical resources among the 31 provinces, $X_{i-base}$ and $X_{i-end}$ represent the medical resources of province i at the base and end of the period, respectively, and n represents the number of provinces.

#### 3.3.2. Spatial Mismatch Index

The spatial mismatch theory, created by Kain, was originally used to analyze the spatial mismatch between housing and employment of disadvantaged groups [74] and is now widely used in human resources, social geography, and tourism economy to measure the degree of mismatch in the geographical distribution of two interrelated resource elements. The spatial mismatch index is used in this paper to analyze whether the geographical distribution of medical resources matches the population and GDP. The calculation equations are shown in (3) to (5). $SMI_{i}$ of zero indicates that the current state of health resources perfectly matches the population demand and economic carrying capacity. $SMI_{i}$ less than zero indicates that the supply of medical resources is insufficient, that is, below the economic carrying capacity and cannot meet the actual demand. $SMI_{i}$ greater than zero indicates an excess supply of health resources, that is, greater than the actual demand and exceeds the economic carrying capacity. A larger absolute value of spatial mismatch index represents a higher degree of mismatch of medical resources with population demand and economic carrying capacity. Due to the complex dynamic and self-adjustment of development, the mismatch of health resources with population and economy will only have a large negative impact on sustainable development when it reaches a certain level. In this paper, the average of positive and negative $SMI_{i}$ values are used as the threshold to classify the spatial mismatch into three types of positive mismatch, dynamic matching, and negative mismatch.

(3) $$SMI_{i}=\frac{X_{i}-\frac{E_{i}}{E}X}{2E}\times 100\%,\;SMI=\frac{\sum_{i=1}^{n}\left|X_{i}-\frac{E_{i}}{E}X\right|}{2E}$$

(4) $$C_{i}=\frac{\left|SMI_{i}\right|}{SMI}\times 100\%$$

where $SMI_{i}$ represents the spatial mismatch index of province i, SMI represents the sum of the absolute values of the study area $SMI_{i}$, $C_{i}$ represents the contribution of the spatial mismatch index, $E_{i}$ represents the demand intensity or supply carrying capacity of province i, measured by two indicators of resident population and GDP, E represents the sum of resident population and GDP of the study area, and X represents the sum of health resources of the study area.

#### 3.3.3. Spatial Econometric Model

This paper analyzes the spatial pattern of health resources and their mismatch by means of spatial clustering analysis, cold and hot spots analysis, and exploratory spatial data analysis (ESDA), which are all data-driven unsupervised learning methods and do not require a priori knowledge. The spatial clustering analysis is conducted by quantile method, and the study area is divided into high, medium, and low levels to analyze the spatial heterogeneity of medical resources. In other words, sort the data in descending order, the top 10 belong to high, 11–20 belong to medium, and 21–31 belong to low. Provinces of the same level share a high degree of similarity, while those at different levels have significant differences. In this paper, regions in statistically significant clusters are identified using the cold and hot spots analysis tool, and the spatial autocorrelation between the evolution trends of health resources and types of mismatches is analyzed using ESDA and characterized using Moran’s I. The global Moran’s I value are in the range of [−1, 1], and a larger absolute value indicates stronger spatial autocorrelation. A value greater than zero indicates spatial positive correlation, less than zero indicates spatial autocorrelation, and equal to zero indicates random distribution [75]. We divide the study area into four types of HH, LL, HL, and LH based on local moran’s I’s LISA map. The first two types represent positive spatial correlation, i.e., a province is similar to its neighbors, while the last two represent negative spatial correlation, i.e., a province is different from or opposite to its neighbors [76,77]. Global and local Moran’s I value are calculated as follows:

(5) $$\mathrm{Global}\;\mathrm{Moran}’s\;I=\frac{n}{S_{0}}\times \frac{\sum_{i=1}^{n}\sum_{j=1}^{n}W_{ij}\left(X_{i}-\bar{X}\right)\left(X_{j}-\bar{X}\right)}{\sum_{i=1}^{n}{\left(X_{i}-\bar{X}\right)}^{2}},\;S_{0}=\sum_{i=1}^{n}\sum_{j=1}^{n}=W_{ij}$$

(6) $$\mathrm{Local}\;\mathrm{Moran}’s\;I_{i}=Z_{i}\sum_{i=1}^{n}W_{ij}Z_{j}$$

where $X_{i}$ and $X_{j}$ represent the attribute values in provinces i and j, respectively, $\bar{X}$ is the average of the $X_{i}$ attribute values, $W_{ij}$ is the spatial weight matrix in global spatial autocorrelation and row normalized value of the spatial weights in local spatial autocorrelation, $S_{0}$ is the sum of the spatial weight matrices, and $Z_{i}$ and $Z_{j}$ are the normalized values of the i and j observations of the study object. The spatial weight matrix is in adjacency mode, and all parameters are default settings. The maximum number of neighbors is 8, and the minimum is 1, mean is 4.45, and median is 4.

#### 3.3.4. Geodetector

Geodetector, created by Wang Jinfeng, a professor at the Institute of Geographic Sciences and Natural Resources Research, Chinese Academy of Sciences, and chief scientist of spatial analysis at the State Key Laboratory of Resource and Environmental Information System, is an emerging statistical analysis method for analyzing spatially driven mechanisms [78,79]. We use Geodetector in this paper to quantitatively measure the influence of different factors on the geographical distribution of health resources and interaction between the factors. Geodetector will assume that the independent variable has a significant influence on the geographical distribution of the dependent variable if the spatial patterns of the independent and dependent variables are similar or even identical. For example, with urbanization and per capita GDP as independent variables and medical resources as a dependent variable, Geodetector measures the similarity of spatial patterns between independent variables and dependent variables by calculating the value of q index based on relying on its factor detection function, characterizing the direct influence of urbanization, and per capita GDP on the geographical distribution of medical resources. The values of q are in the range of [0,1], and a larger value indicates that it has a greater influence. With h representing the number of strata or classifications of the independent variables, $N_{h}$ and N representing the number of cities in stratum h and the study area, with $\sigma_{h}^{2}$ and $\sigma^{2}$ representing the variance of the dependent variable in stratum h and the study area, respectively, SSW representing the within sum of squares, and SST representing the total sum of squares in the study area, q is calculated as follows:

(7) $$q=1-\frac{\sum_{h=1}^{l}N_{h}\sigma_{h}^{2}}{N\sigma^{2}}=1-\frac{SSW}{SST},\;SSW=\sum_{h=1}^{l}N_{h}\sigma_{h}^{2},\;SST=N\sigma^{2}$$

Notably, the interaction detection function of Geodetector enables further measurement of the interactive influence of urbanization and per capita GDP when they act together on the geographical distribution of health resources. With *q*($X_{i}$) and *q*($X_{j}$) representing the direct influence with the two factors i and j in the independent case, *q*($X_{i}$∩$X_{j}$) representing the interaction influence of the two factors i and j in the joint action, Max(*q*($X_{i}$)), *q*($X_{j}$)) and Min(*q*($X_{i}$), *q*($X_{j}$)), *q*($X_{i}$) + *q*($X_{j}$) representing the maximum, minimum, and sum of the direct influence of the two factors i and j in the independent case, the factor interactions are classified into five types, based on the relationship of the aforementioned parameters. Where, when *q*($X_{i}$∩$X_{j}$) < Min(*q*($X_{i}$), *q*($X_{j}$)), it means that the factors i and j inhibit each other in an antagonistic state, which is defined as nonlinear weaken. When Min(*q*($X_{i}$), *q*($X_{j}$)) < *q*($X_{i}$∩$X_{j}$) < Max(*q*($X_{i}$)), *q*($X_{j}$)), it means that the interaction influence lies between the maximum and minimum values of direct influence, which is defined as single nonlinear weaken. When *q*($X_{i}$) + *q*($X_{j}$) > *q*($X_{i}$∩$X_{j}$) > Max(*q*($X_{i}$), *q*($X_{j}$)), it means that the interaction influence is greater than the maximum value of the direct influence in the independent case, but less than the sum of the two, which is defined as bifactor enhancement. When *q*($X_{i}$∩$X_{j}$) = *q*($X_{i}$) + *q*($X_{j}$), it means that the influence of two factors are independent of each other, which is defined as independent. When *q*($X_{i}$∩$X_{j}$) > *q*($X_{i}$) + *q*($X_{j}$), it indicates that the factors i and j are synergistically enhanced with each other, which is defined as nonlinear enhancement [80].

## 4. Results

### 4.1. Spatio-Temporal Dynamics

#### 4.1.1. Spatial Pattern

**Figure 4:**
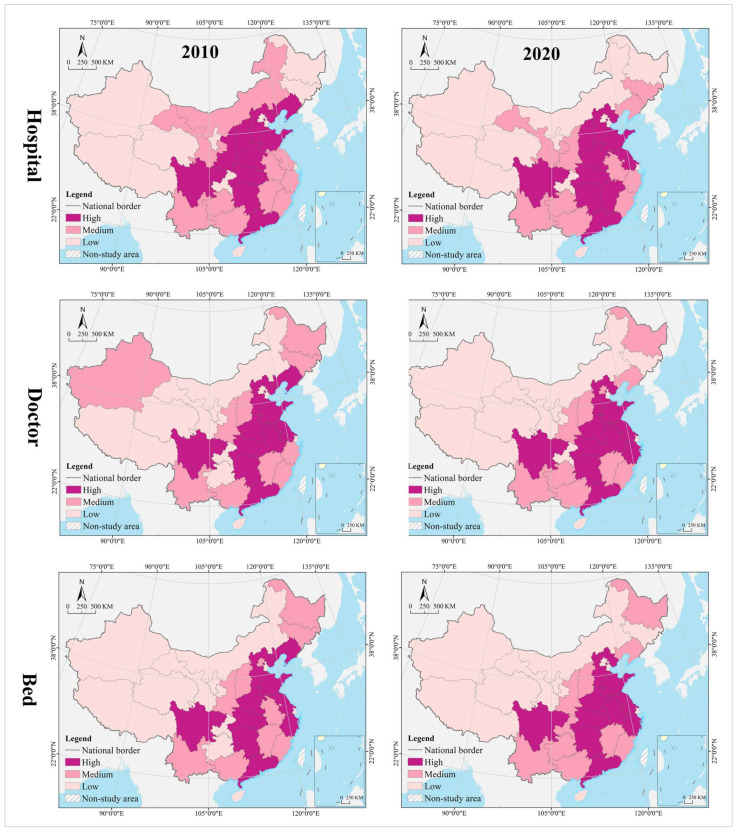
Distribution pattern of medical resources.

The spatial pattern of geographical distribution of medical resources has been stable for a long time, with hospitals, doctors, and beds in spatial clustering patterns that are nearly the same, and the spatial heterogeneity level within a reasonable range. In 2010, the thresholds for spatial cluster analysis of hospitals, doctors, and beds were 34,269 (Hubei) and 22,565 (Inner Mongolia), 316,828 (Liaoning) and 187,106 (Jilin), and 188,100 (Zhejiang) and 113,000 (Fujian), respectively. In 2020, their thresholds are 35,447 (Hubei) and 25,616 (Jilin), 503,172 (Anhui) and 310,391 (Heilongjiang), and 361,300 (Zhejiang) and 216,800 (Fujian), respectively. In 2010 and 2020, the provinces of high level formed an “X” shaped agglomeration zone along Guangdong-Hebei and Sichuan-Jiangsu, including Guangdong, Hunan, Jiangxi, Hubei, Henan, Shandong, Shanxi, and Hebei. Provinces of medium level were distributed at the edge of the high-level zones, clustered in the Loess Plateau, Yunnan-Guizhou Plateau, Beibu Gulf, West Coast of the Strait, Yangtze River Delta, and northeast China. Most of the provinces of low level were clustered in northwest China, including Tibet, Qinghai, Xinjiang, Inner Mongolia, and Gansu (Figure 4). The Gini indices for hospitals, doctors, and beds in 2010 and 2020 were 0.40, 0.39, 0.36, 0.36, 0.35, and 0.37, respectively, all of which were not greater than 0.40, with a narrow change range.

#### 4.1.2. Change Process

**Figure 5:**
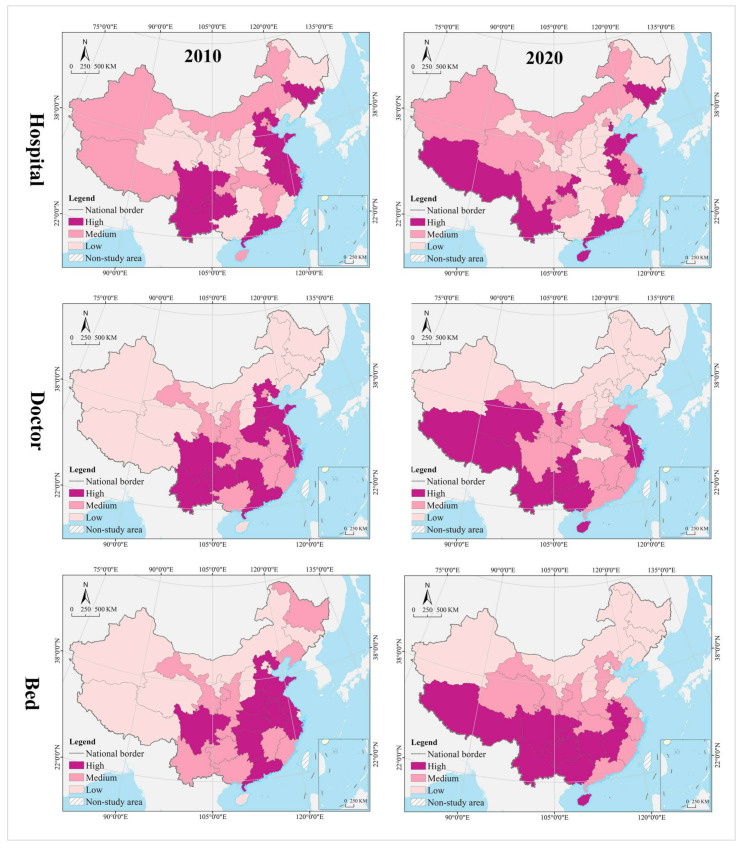
Distribution pattern of medical resources change.

The change range of medical resources was characterized by clustering, and the spatial pattern of hospitals, doctors, and beds varied widely. Using the GIS spatial clustering quantile analysis tool, the change range of hospitals, doctors, and beds in 31 provinces from 2010 to 2020 were divided into three categories—high, medium, and low. The thresholds were 3460 (Guizhou) and 1178 (Hubei), respectively. 212,640 (Guizhou) and 89,469 (Shanghai), 177,200 (Zhejiang) and 81,500 (Gansu). From the perspective of hospitals, the high-level provinces were clustered in the eastern coastal regions and the Yunnan-Guizhou Plateau in the southwest; the medium-level provinces were distributed in a zonal pattern along the western and northern borders, while the low-level provinces were mostly clustered in the central, central south, and Loess Plateau regions. Shandong had the largest number of new hospitals (17,905), compared to the largest number of hospital reductions (−3317) in Hunan, and Liaoning, Heilongjiang, Henan, Shaanxi, and Gansu provinces saw varying degrees of reductions in the number of hospitals. It is worth noting that the northeast and northwest regions, such as Liaoning, Heilongjiang, and Gansu not only have serious population loss problems, but also experienced negative economic growth. From 2010 to 2020, their hospital reduction, doctors, and beds increase, whether this complex change phenomenon is reasonable needs to be further analyzed by other methods (spatial dislocation part). From the perspective of hospitals, the high-level provinces were clustered in the eastern coastal regions and Pan-Pearl River Delta urban agglomeration; the medium-level provinces were distributed in the middle of the two high-level clusters, and the low-level provinces were in north (including northeast and northwest) and west China. The largest number of new doctors was in Guangdong (413,426), while the smallest was in Tibet (24,333). From the perspective of beds, the high-level provinces covered most of the eastern and central regions, the medium-level provinces were distributed in the periphery of those of high level, mostly clustered in the Yunnan-Guizhou Plateau in the southwest and Loess Plateau in the northwest, and the low-level provinces were clustered in the west and north. The largest increase in beds was in Sichuan (348,500), while the smallest was in Tibet (90,700) (Figure 5).

The change speed of medical resources showed gradient agglomeration, and the high-middle-low level areas shaped a center-periphery spatial structure in geographical distribution. Using the GIS spatial clustering quantile analysis tool, the change speed of hospitals, doctors, and beds in 31 provinces from 2010 to 2020 were divided into three categories, i.e., high, medium, and low, with thresholds of 1.52% (Yunnan) and 0.75% (Jiangxi), 5.90 (Guangxi) and 4.75 (Henan), and 7.48% (Guangxi) and 5.87% (Hebei), respectively. In terms of hospitals, the high-level regions were scattered in distribution, including Guangdong, Hainan, Chongqing, Yunnan, Tibet, Shandong, Tianjin, Shanghai, Anhui, and Jilin. The medium-level provinces were on the periphery of those of high level, mostly clustered in west and north China. Most of the low-level provinces were clustered in central and south-central China, the Loess Plateau, and the Bohai Bay. Tibet recorded the largest average annual growth (3.41%), while Heilongjiang recorded the largest decline (−0.76%). The average of change speed for hospital was 1.14%, with 48.39% of provinces exceeding it. In terms of doctors, the high-level provinces were mostly clustered in the Yunnan-Guizhou Plateau in the southwest and the Qinghai-Tibet Plateau; the medium-level provinces were distributed in the periphery of those of high level, and the low-level provinces were distributed in north China (especially in northern and northeast China). The largest average annual growth of doctors was in Tibet (9.41%), and the smallest was in Heilongjiang (1.69%). The average of change speed for doctor was 5.26%, with 45.16% of provinces exceeding it. In terms of beds, the provinces of high, medium and low level were clustered in a gradient distribution along the north–south direction, with the largest annual growth in Guizhou (10.13%) and smallest in Beijing (3.19%). The average of change speed for bed was 6.16%, with 61.29% of provinces exceeding it.

#### 4.1.3. Evolution Trend

**Figure 6:**
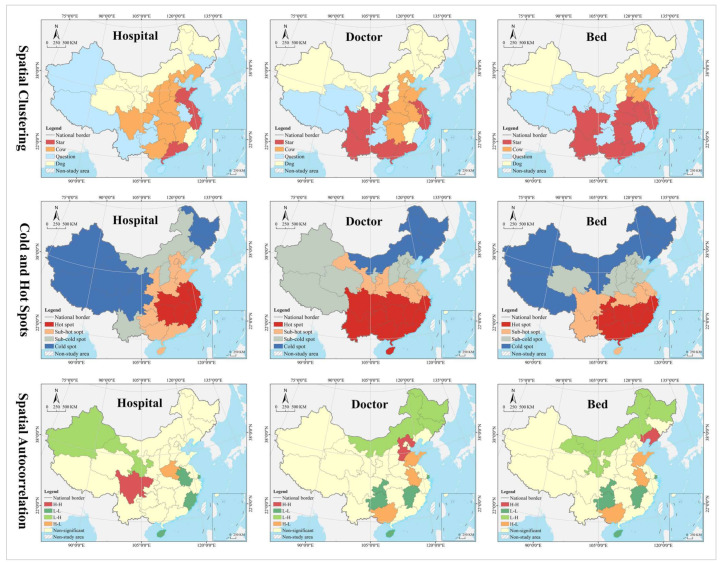
Spatial analysis of evolution trend.

In the hospital dimension, star provinces were clustered in the eastern coastal regions, cow provinces were in the central region, question provinces were mostly clustered along the western border, and dog provinces were clustered in north China. The hot and cold spots were clustered along the east–west direction in a gradient manner. Hot spot provinces were clustered in the middle and lower reaches of the Yangtze River, with secondary hot spots clustered in its periphery. Cold spot provinces were clustered in the northwest and northeast, with secondary cold spots clustered in north China, including Inner Mongolia, Shanxi, and Liaoning. Global Moran’s I was 0.14, indicating a positive spatial autocorrelation. According to local autocorrelation analysis, HH- and HL-type provinces were only Sichuan and Henan, LL-type provinces included Hainan, Anhui, Fujian, and Shanghai, and LH-type regions included Gansu and Xinjiang (Table 2 and Figure 6).

In the doctor dimension, most of the star provinces were clustered in southwest China, including Yunnan, Guangxi, Guangdong, Sichuan, Shaanxi, Jiangsu, and Zhejiang. The cow provinces were clustered in the central China and extended to Hebei and Shandong. The question provinces were scattered in distribution, including Hunan, Chongqing, Ningxia, Qinghai, and Tibet. Most of the dog provinces were clustered in north China, especially in the northeastern region. The hot and cold spots were clustered along the north–south direction in a gradient manner. Hot spot provinces were clustered in the Yangtze River Economic Belt and its south, with secondary hot spots distributed in its periphery in the Yellow River Economic Belt. Cold spots were clustered in the northeast, with secondary cold spots clustered in the west, Beijing, Tianjin, and Hebei and their surrounding areas. Global Moran’s I was 0.15, indicating a positive spatial autocorrelation. According to the local autocorrelation analysis, only Hebei was an HH-type region; HL-type provinces included Guangxi, Anhui, and Shandong, LL-type regions included Hainan, Jiangxi, Chongqing and Guizhou, and LH--type regions included Inner Mongolia, Heilongjiang and Jilin.

In the bed dimension, the star provinces were clustered in the Yangtze River Delta and Pan-Pearl River Delta urban agglomeration, the cow provinces were clustered in the Bohai Bay, the question provinces were clustered in Qinghai-Tibet and Loess Plateau regions and the west coast of the strait, and the dog provinces were clustered in north China. The hot and cold spots were clustered along the north–south direction in a gradient manner. Hot spot was clustered in clustered in south China, with secondary hot spots clustered in its periphery. The cold spot provinces were clustered along the border in west and north China, with secondary cold spots clustered in the middle and lower reaches of the Yellow River Basin and the Beijing-Tianjin-Hebei region. Global Moran’s I was 0.23, indicating a positive spatial autocorrelation. According to the local autocorrelation analysis, only Liaoning was an HH-type province, HL-type provinces included Guangxi, Anhui and Shandong, LL-type provinces included Hainan, Jiangxi, Chongqing and Guizhou, and LH-type provinces included Gansu, Inner Mongolia, and Heilongjiang.

The analysis of spatio-temporal evolution trend based on Boston Consulting Group Matrix contributes to revealing the change trend of health resource allocation, for the purpose of developing differentiated and adaptive management strategies for different provinces. The strategies for future health resource supply include three types of development, stability, and retrenchment. The development strategy is to invest in additional health resources, expand the medical resource supply, and improve health services. The stabilization strategy is to stabilize the current supply mode and allocation status without additional investment, try to maximize the value, and efficiently use of health resources. The retrenchment strategy is to scale back the supply of health resources and address the problems of waste or extensive use. In general, star provinces should adopt development strategy, cow provinces should adopt stabilization strategy, question provinces can selectively implement development, stabilization or retrenchment strategy based on their actuality, and dog provinces can selectively adopt retrenchment or stabilization strategy. It should be noted that all provinces should choose the most appropriate strategy to maintain or reverse the development pattern or trend, according to the health resource supply and demand balance (mismatch or adaptation) (analysis is made in the discussion section based on the spatial mismatch calculation results).

### 4.2. Spatial Mismatch Analysis

#### 4.2.1. Demand: Population Potential Consumption

(1) Spatial Mismatch Type of Population

**Figure 7:**
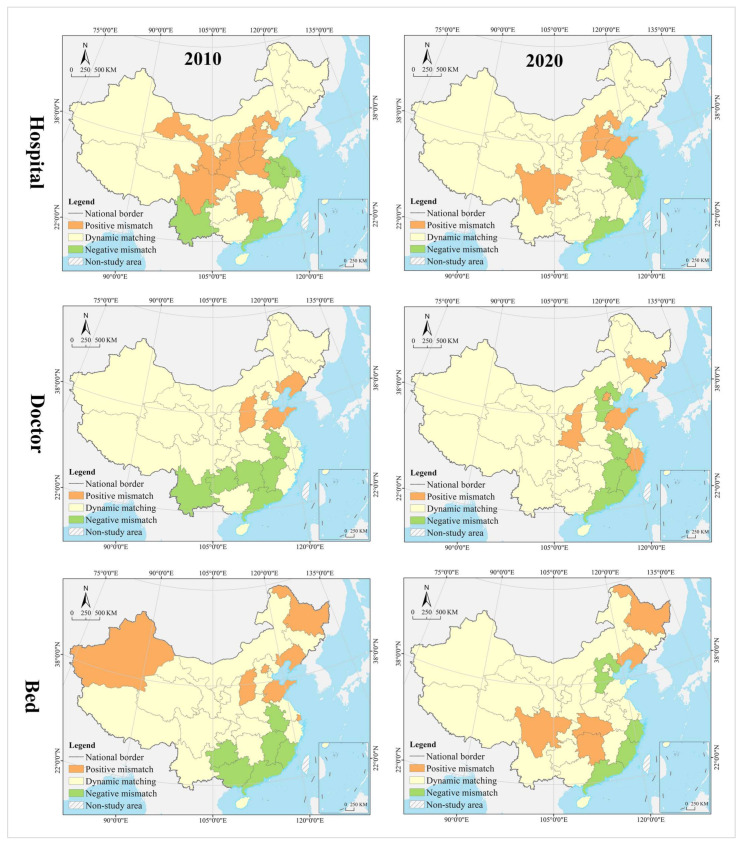
Spatial clustering analysis of mismatch type for demand.

**Figure 8:**
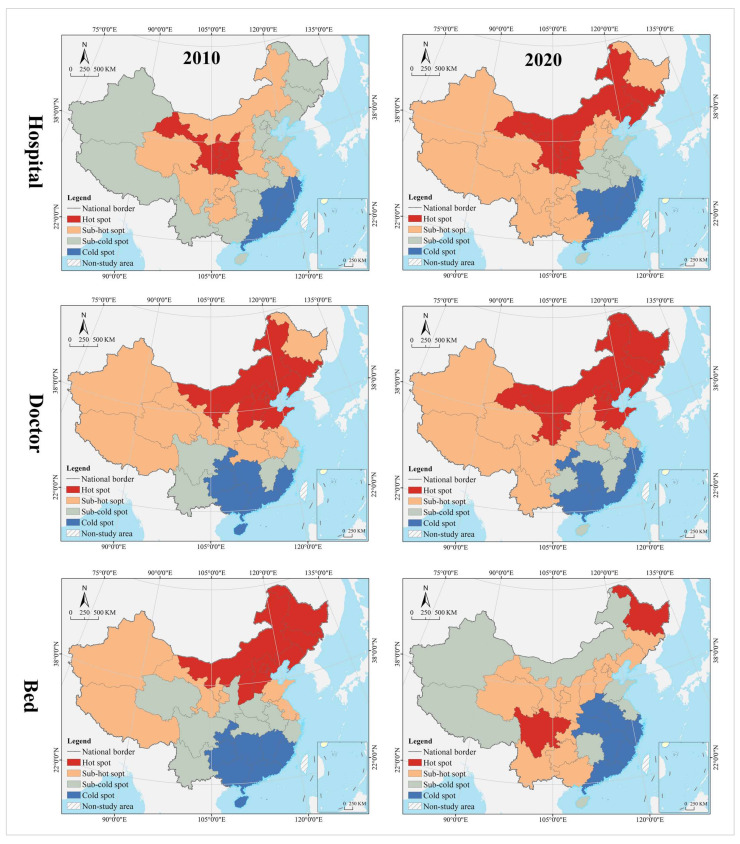
Spatial cold and hot spots analysis of mismatch type for demand.

**Figure 9:**
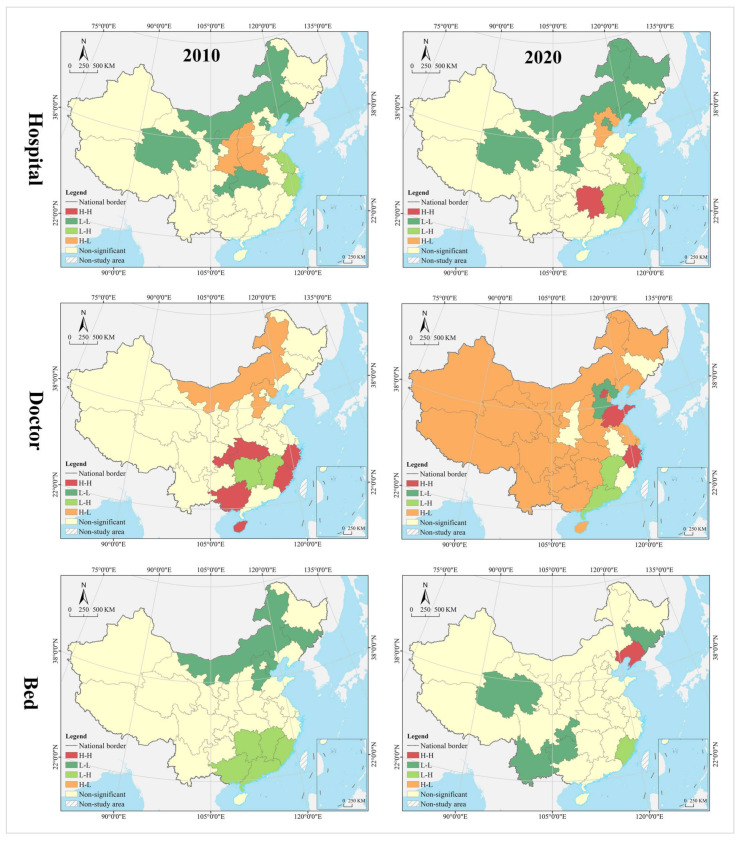
Spatial autocorrelation analysis of mismatch type for demand.

In the hospital dimension, 61.29% of the provinces fell into the dynamic matching type in 2010, further expanding to 70.97% in 2020. In 2010, 22.58% of the provinces were of the positive mismatch type, distributed in the middle and upper reaches of the Yellow River Basin in clusters; by 2020 the cluster area shrank significantly, with most of them in the lower reaches of the Yellow River Basin. There were the same number of provinces belonging to the negative mismatched type in 2010 and 2020, but their geographical distribution was changed from random to agglomerative (Yangtze River Delta urban agglomeration) (Figure 7). In 2010, the hot spots were clustered in the Loess Plateau, with secondary hot spots distributed in its periphery in a ring pattern. The cold spots were clustered along the southeast coast, with secondary cold spots clustered along the western, southwestern and northeastern borders and extending to the south-central region and the Bohai Bay. In 2020, the hot spots were clustered in the Loess Plateau and extended to the northeast, with most of the secondary hot spots clustered in the west. The cold spots were clustered in the Pearl River Delta and its surrounding areas, with secondary cold spots clustered in the Yangtze River Delta and its surrounding areas (Figure 8). The global Moran’s I value for 2010 and 2020 were 0.21 and 0.28, respectively, indicating a positive spatial autocorrelation. In the dimension of local spatial autocorrelation, there was no HH-type region in 2010, but only Hunan in 2020. The LL-type regions have long been clustered in north China, including Inner Mongolia, Liaoning, Tianjin, Beijing, and Qinghai. The HL-type regions were clustered in the middle reaches of the Yellow River in 2010 and shank to only Hebei in 2020. The LH-type regions were clustered in the Yangtze River Delta in 2010 and extended southward to the west coast of the Strait in 2020 (Figure 9).

In the doctor dimension, 67.74% of provinces fell into the dynamic matching type in both 2010 and 2020, covering the western, northern, and central regions of China. In 2010, 12.9% of the provinces were of the positive mismatch type and relatively concentrated in the Bohai Bay, compared to a random distribution in 2020, including Jilin, Beijing, Shandong, Shanxi, and Zhejiang. Provinces of the negative mismatch type in 2010 shaped a belt-like agglomeration in the Pan-Pearl River Delta in the east–west direction and changed to a belt-like agglomeration in the north–south direction in 2020. In 2010, the hot spots were clustered in north China and the Bohai Bay, with secondary hot spots clustered in their western and southern periphery. The cold spots were clustered in the Pan-Pearl River Delta urban agglomeration, with secondary cold spots only including Yunnan, Sichuan, Jiangxi, and Zhejiang. In 2020, the hot spots covered north China and northeast China and extended to the Loess Plateau in northwest China, with secondary hotspots clustered in the west. The cold spots were clustered on the southeast coast and extended to Hunan and Chongqing, with secondary cold spots clustered in central China, including Anhui, Jiangxi, and Hubei. The global Moran’s I value for 2010 and 2020 were 0.15 and −0.10 *, respectively (* represents *p* > 0.05, the same below), indicating a shift from positive spatial autocorrelation to no correlation. In the dimension of local spatial autocorrelation, Zhejiang, Fujian, Chongqing, Hubei, Guangxi, and Hainan were HH-type regions in 2010, which were changed to Shandong, Beijing, and Zhejiang in 2020. Inner Mongolia and Hebei were HL-type provinces in 2010, which were expanded to most parts of the country in 2020. Provinces of LH- and LL-types were scarce and distributed in a random manner, including Hunan, Jiangxi, Guangdong, and Hebei.

In the bed dimension, 58.06% of the provinces fell into the dynamic matching type in 2010, further expanding to 70.97% in 2020. In 2010, 22.58% of the provinces fell into the positive mismatch type, a figure that contracted to 16.13% in 2020 in a random distribution, with the former including Xinjiang, Shanghai, Beijing, Shandong, Shanxi, Liaoning, and Heilongjiang, and the latter including Sichuan, Hunan, Hubei, Liaoning, and Heilongjiang. In 2010, 19.35% of the provinces were in negative mismatch, clustered in “crescent-shaped” distribution, including Guizhou, Guangxi, Guangdong, Fujian, Jiangxi, and Anhui. The geographic coverage in 2020 shrank rapidly, mostly clustered in the southeast coastal area. The hot spots in 2010 covered north and northeast China, with most of the secondary hot spots distributed in the western border regions. The cold spots were clustered in south China, with the secondary cold spots distributed in its periphery. In 2020, the hot spots shrank rapidly to only Heilongjiang and Sichuan, with the secondary hot spots clustered in “Y” shaped distribution in the middle. The cold spots were clustered in the middle reaches of the Yangtze River, as well as the west coast of the Strait and Great Bay area, and most of the secondary cold spots were distributed in a band along the western and northern borders. The global Moran’s I values for 2010 and 2020 were 0.18 and 0.07 *, respectively, indicating a shift from positive spatial autocorrelation to no correlation. In the dimension of the local spatial autocorrelation, the HH- and HL-type regions were poorly developed. The LL-type regions were clustered in north China in 2010 and shifted to southwest China in 2020. The LH-type regions were clustered in the Pan-Pearl River Delta in 2010 and shrank to only Fujian in 2020.

(2) Mismatch Index Contribution Rate of Population

**Figure 10:**
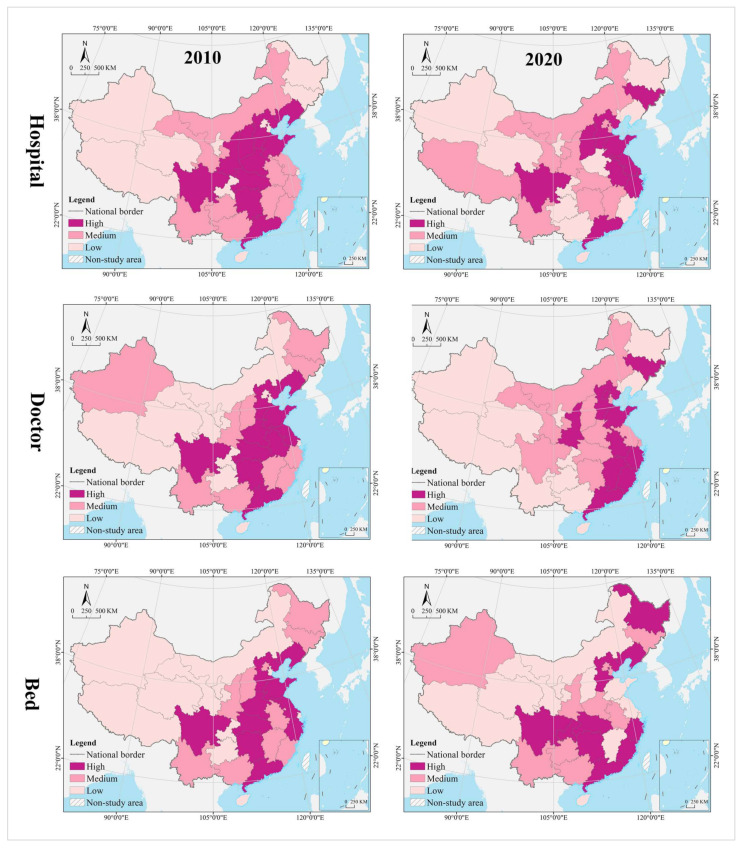
Spatial analysis of mismatch index contribution rate for demand.

In 2010, the provinces with higher contribution to hospital mismatch were clustered in the center of China in a “T” shape, while the provinces of medium level were distributed in the periphery of the high level, and those of low level were distributed in the more peripheral northwest and northeast regions. High-medium-low level provinces shaped a closer center-periphery spatial structure in geographical distribution. In 2020, most of the provinces of high level were clustered in the eastern coast, the lower reaches of the Yangtze and Yellow rivers, with Jilin, Sichuan, and Shandong being isolated spots in the periphery. Provinces of medium level were clustered in the middle reaches of the Yangtze River and middle and upper reaches of the Yellow River, and those of low level were scattered in distribution, including Xinjiang, Qinghai, Ningxia, Heilongjiang, Liaoning, Fujian, Henan, Chongqing, Sichuan, Guangxi, and Hainan (Figure 10).

In 2010, the provinces with higher contribution to doctor mismatch were clustered along Guangdong-Hubei-Liaoning in a north–south belt, and those of medium level were distributed in its periphery, including Fujian, Jiangxi, Zhejiang, Heilongjiang, Jilin, Shanxi, Shaanxi, Guangxi, Hainan, and Yunnan. Provinces of low level were clustered along the Loess Plateau–Qinghai-Tibet Plateau direction, including Gansu, Ningxia, Qinghai, Tibet, and Inner Mongolia. In 2020, three types of regions showed an east–west gradient distribution. Provinces of high level were still in the north–south belt-like agglomeration, but their regional coverage began to shrink, with those of medium level clustered in the middle area, and those of low level in the northwest and southwest China.

In 2010, the provinces with higher contribution to bed mismatch were clustered in the central and coastal regions in an “X” shape, with those of medium of distributed in their periphery, as well as those of low level clustered in northwest China. Provinces of high level in 2020 were distributed in a band in the middle and upper reaches of the Yangtze River and the southeast coast, including Sichuan, Chongqing, Hubei, Hunan, Shandong, Fujian, Zhejiang, Hebei, and Liaoning. Most of the provinces of medium were clustered in the north and south of the high level, including Yunnan, Guizhou, and Guangxi in the southwest and Shaanxi, Henan, and Anhui in the central part of the country. Provinces of low level were mostly clustered in the Qinghai-Tibet Plateau and Loess Plateau, with a small number randomly distributed in the coastal areas, including Shandong and Jiangsu.

#### 4.2.2. Supply: Economic Carrying Capacity

(1) Spatial Mismatch Type of GDP

**Figure 11:**
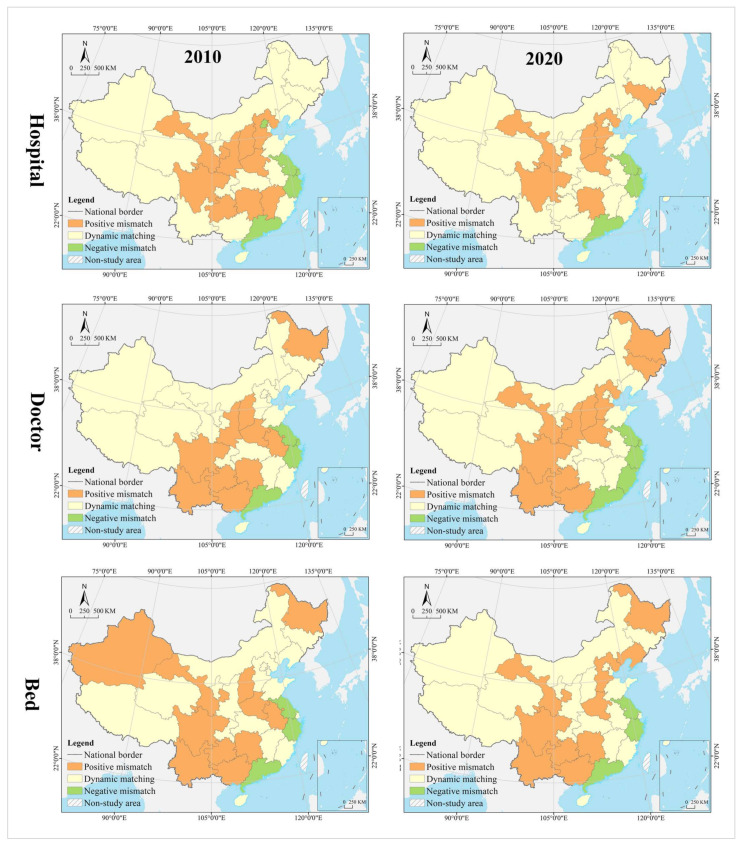
Spatial clustering analysis of mismatch type for supply.

**Figure 12:**
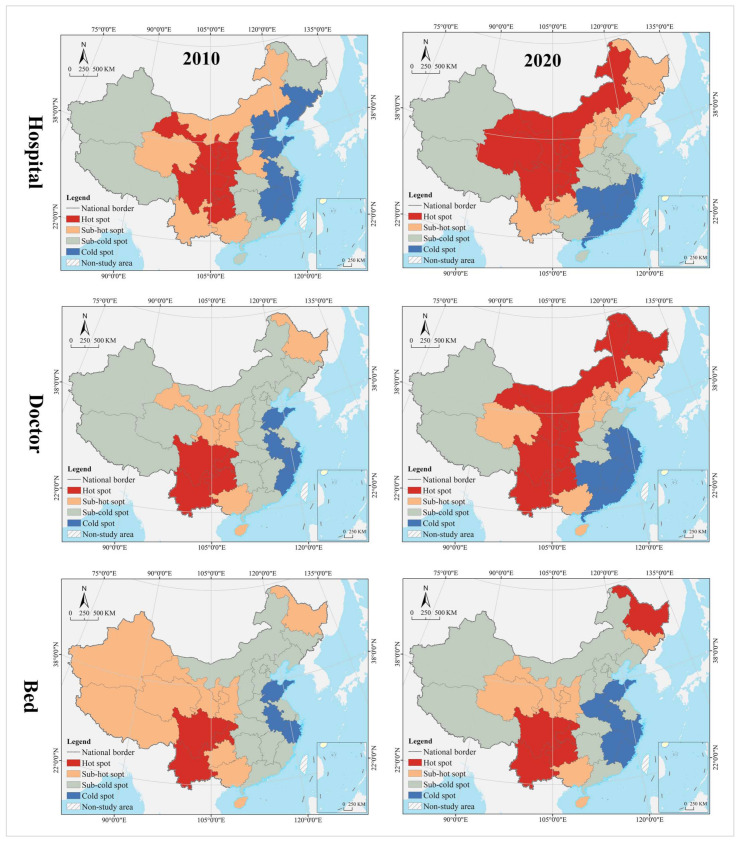
Spatial cold and hot spots analysis of mismatch type for supply.

**Figure 13:**
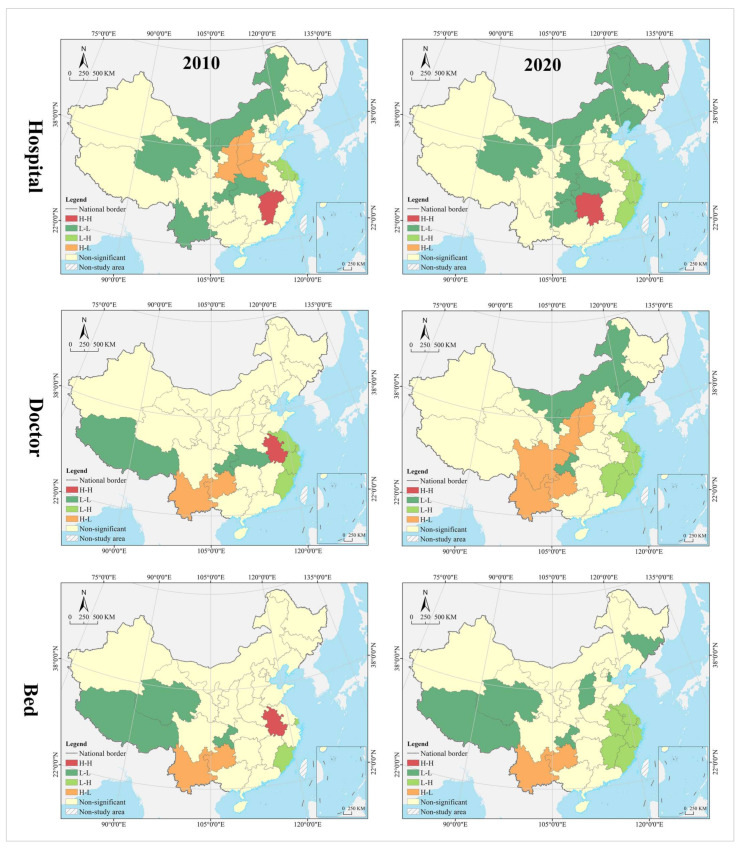
Spatial autocorrelation analysis of mismatch type for supply.

In the hospital dimension, 54.84% of the provinces fell into the dynamic matching type in 2010, further expanding to 64.52% in 2020. In 2010, 29.03% of the provinces fell into the positive mismatch type, mostly clustered in the middle and upper reaches of the Yellow River and middle reaches of the Yangtze River, but the cluster area shrank significantly to only the east and west ends by 2020. Provinces that fell into the negative mismatch type in 2010 and 2020 were essentially equivalent (about 15%), including Jiangsu, Zhejiang, and Guangdong (Figure 11). In 2010, the hot spots were clustered in the Loess Plateau, with secondary hot spots scattered in its periphery. The cold spots were clustered in the coastal regions of east and north China, with secondary cold spots mostly clustered along the northwest and northeast borders. In 2020, the hot spots were clustered in the Loess Plateau and extended to the northeast, with secondary hot spots scattered in its periphery. The cold spots were clustered in the southeast coast, with secondary cold spots clustered in the Yangtze River Delta, the Beibu Gulf, and west China (Figure 12). The global Moran’s I value for 2010 and 2020 were 0.23 and 0.24, respectively, indicating a positive spatial autocorrelation in a stable state. In the dimension of local spatial autocorrelation, there were a small number of HH- and HL-type regions, changing from Jiangxi, Shanxi, and Shaanxi in 2010 to Hunan and Hebei in 2020. In 2010, the LL-type regions were Inner Mongolia, Qinghai, Chongqing, Hubei, Yunnan, and Guangdong, but changed to Inner Mongolia, Shaanxi, Qinghai, Liaoning, and Heilongjiang in 2020, which were increasingly concentrated in the north, especially in the northeast. The LH-type regions have long been clustered in the Yangtze River Delta and are gradually expanded to the west coast of the Strait (Figure 13).

In the doctor dimension, about 50% of provinces fell into the dynamic matching type in both 2010 and 2020, with contraction in the north and expansion in the center. The same percentage of provinces were seen falling into the positive mismatch type in 2010 and 2020 (35.48%), mostly clustered in the Yunnan-Guizhou Plateau and Yellow River Basin. Provinces of the negative mismatch type in 2010 and 2020 were equivalent, clustered along the coast in a band. In 2010, the hot spots were clustered in the Yunnan-Guizhou Plateau in southwest China, with secondary hot spots clustered in the Loess Plateau and Beibu Gulf. The cold spots were clustered in the Shandong-Anhui-Zhejiang-Fujian region, with cold spots widely distributed. In 2020, the hot spots were clustered in the Yunnan-Guizhou Plateau–Loess Plateau-northeast direction, with secondary cold spots mostly in Bohai Bay. The cold spots were clustered in the Pearl River Delta–Yangtze River Delta region, with secondary cold spots clustered in the lower reaches of the Yellow River and west China (Tibet and Xinjiang). The global Moran’s I value for 2010 and 2020 were 0.30 and 0.46, respectively, indicating a positive spatial autocorrelation with increasing strength. In the dimension of local spatial autocorrelation, Anhui fell into the HH-type in 2010, and no HH-type region was found in 2020. Provinces of HL-type were Yunnan and Guizhou in 2010, but expanded to Sichuan, Shaanxi, and Shanxi in 2020. In 2010, Tibet, Chongqing, and Hubei were LL-type regions, which were changed to Inner Mongolia, Liaoning, and Chongqing in 2020. The LH-type regions were clustered in the eastern coast, including Jiangsu, Shanghai, Zhejiang, and Fujian.

In the bed dimension, 54.84% of the provinces fell into the dynamic matching type in 2010, expanding slightly to 58.06% in 2020, mostly clustered in west, north, and central China. About 35% of the provinces falling into the positive mismatch type in both 2010 and 2020, mostly clustered in the Yunnan-Guizhou Plateau and its surrounding areas. The regions that fell into the negative mismatch type were exactly the same in 2010 and 2020, including Jiangsu, Zhejiang, and Guangdong. In 2010, the hot spots were clustered in the Yunnan-Guizhou Plateau, with secondary hot spots clustered in the west of its periphery, the Loess Plateau, and Beibu Gulf. The cold spots included Zhejiang, Anhui, and Shandong, with secondary cold spots distributed in north, central, and south China as transitional zones. In 2020, the hot spots were still clustered in the Yunnan-Guizhou Plateau, with secondary hot spots clustered in the Loess Plateau and Beibu Gulf. The cold spots were clustered in the Yellow River and lower reaches of the Yangtze River, with secondary cold spots clustered in northwest, north, and central China. The global Moran’s I for 2010 and 2020 were 0.15 * and 0.19, respectively, indicating a shift from spatial uncorrelation to positive autocorrelation. In the dimension of local spatial autocorrelation, only Anhui fell into the HH-type in 2010, and no HH-type region was found in 2020. In 2010, Tibet, Qinghai, and Chongqing fell into the LL-type, which was further expanded to Shanxi, Tianjin, and Jilin in 2020. Yunnan and Guizhou maintained their status as HL-type regions in 2010 and 2020. The LH-type regions were Shanghai and Fujian in 2010, which were expanded to the Yangtze River Delta and west coast of the strait in 2020.

(2) Mismatch Index Contribution Rate of GDP

**Figure 14:**
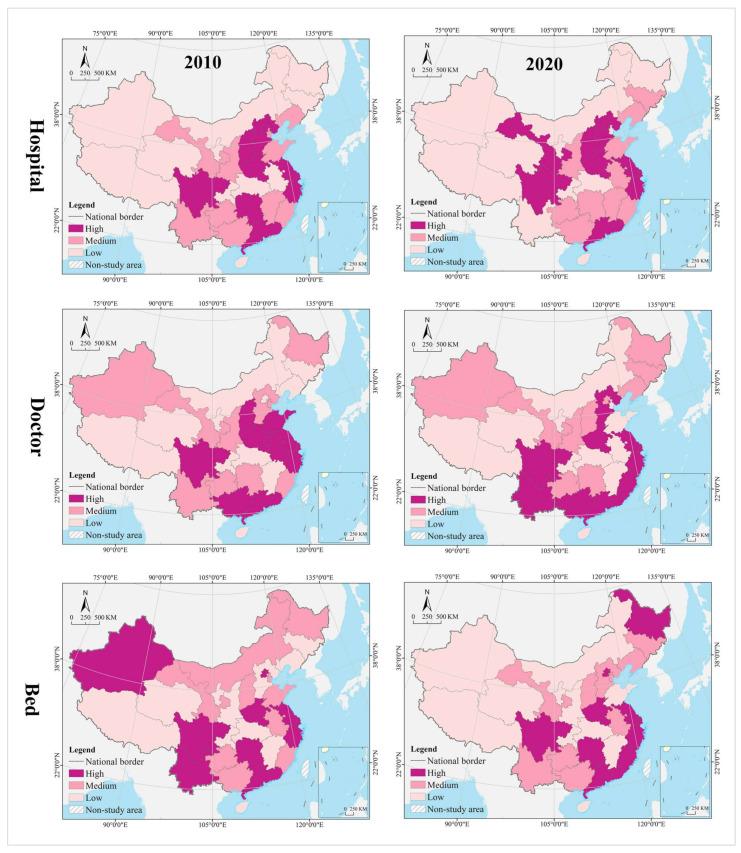
Spatial analysis of mismatch index contribution rate for supply.

In 2010 and 2020, the provinces with high contribution to hospital mismatch were clustered in the Yangtze River Delta, Pearl River Delta, and upper and lower reaches of the Yellow River, with those of medium level distributed in their periphery, low level clustered in west and north China, and middle reaches of the Yangtze River Economic Belt. In 2010, the provinces with high contribution to doctor mismatch were clustered in the lower reaches of the Yangtze River, Yellow River, and south China, with those of medium level distributed in the periphery and extending northwest; those of low level clustered in the Qinghai-Tibet Plateau, the middle reaches of the Yangtze River, and northeast China. Provinces of high level in 2020 shaped a coastal agglomeration in the east and extended north to Henan and Hebei and west to Yunnan and Sichuan. Provinces of medium level were clustered in northeast and northwest China, with those of low level clustered in the Qinghai-Tibet Plateau and middle reaches of the Yellow River. The provinces with high contribution to bed mismatch in 2010 were scattered in distribution, including Jiangsu, Zhejiang, Henan, Beijing, Guangdong, Hunan, Yunnan, Sichuan, and Xinjiang. Provinces of medium level were relatively clustered in north China and Beibu Gulf, with those of low level clustered in the Qinghai-Tibet Plateau, central China and Bohai Bay. In 2020, most of the provinces of high level were clustered in a belt-like pattern along the coast, with those of medium level clustered in southwest China and Bohai Bay, and those of low level clustered in the west, north, and central China (Figure 14).

### 4.3. Driving Mechanism

#### 4.3.1. Influence Factor

The driving forces of influence factors on hospitals, doctors, and beds differed significantly, with the mean values of 0.52, 0.79, and 0.72, respectively (*p* < 0.05). With the mean value as the threshold, the influence factor with a force greater than the mean is defined as the key factor; the one smaller than the mean is defined as an important factor, and the one not statistically significant is defined as an auxiliary factor.

The influence of the children and elderly population, health care government investment, and service industry added value on the geographic distribution of hospitals is greater than the mean, especially children and elderly population, which is more influential than other factors as a key factor. The influence of government revenue, high quality population, and medical insurance fund expenditure is less than the mean, but cannot be ignored as an important factor. Social consumption, residents’ medical services consumption, per capita GDP and urbanization rate are less influential and not statistically significant as auxiliary factors (Table 3).

The influence of the children and elderly population, health care government investment, and social consumption on the geographic distribution of doctors is greater than the mean, especially, the influence of children and elderly population is more than 0.9 as a key factor. The influence of service industry added value, high quality population, medical insurance fund expenditure, and government revenue is less than the mean as an important factor. The influence of per capita GDP, urbanization rate and residents’ medical services consumption are not statistically significant and belong to cofactors.

The children and elderly population, health care government investment, social consumption are key factors influencing the geographic distribution of beds. Service industry added value, high quality population, medical insurance fund expenditure, and government revenue are important factors. Per capita GDP, urbanization rate and residents’ medical services consumption are auxiliary factors.

#### 4.3.2. Interaction Effect

The interaction between the influence factors is dominated by bifactor enhancement, with more than 30% of the factor pairs in the hospital, doctor, and bed dimensions being of nonlinear enhancement. Although the factors per capita GDP and urbanization rate have a direct influence that is not statistically significant, they have strong nonlinear enhancement effects when acting together with other factors. The interaction influence of impact factors on hospitals is generally low, with only $X_{1}$∩$X_{6}$, $X_{2}$∩$X_{6}$, $X_{3}$∩$X_{6}$*,* $X_{4}$∩$X_{6}$, $X_{5}$∩$X_{6}$, $X_{8}$∩$X_{6}$, and $X_{10}$∩$X_{6}$ having an interaction influence greater than 0.85. Per capita GDP, the urbanization rate and children and elderly population generally have higher influence when they interact with other factors, and they can be regarded as super interaction factors. The interaction influence of influence factors on doctors and beds is generally high, with about 50% of factor pairs having an interaction influence greater than 0.9. In particular, the interaction influence of factor pairs, such as $X_{1}$∩$X_{6}$, $X_{2}$∩$X_{6}$, $X_{3}$∩$X_{6}$, $X_{4}$∩$X_{6}$, $X_{5}$∩$X_{6}$, $X_{7}$∩$X_{6}$,$X_{8}$∩$X_{6}$, and $X_{10}$∩$X_{6}$, is greater than 0.95 (Table 4, Table 5 and Table 6).

## 5. Discussion

### 5.1. Extended Thinking and External Evidence

Inequity in the distribution and allocation of medical resources among different regions remains a worldwide problem [81,82], and China is no exception [83,84]. Some of the analytical results of this study reconfirm the conclusions of some scholars, such as the spatial heterogeneity [85], agglomeration [86], autocorrelation and correlation [87,88], non-equilibrium [89], and inequality [90] in the geographic distribution of medical resources and their spatio-temporal evolution in China; additionally, the geographic inequality of hospitals is greater than that of doctors and beds [91]. Some of the analytical results in this paper are not exactly the same or even contrary to the current knowledge and conclusions, and they are of enlightening value. Zhu [92] proposed to address the uneven geographical distribution of doctors in China from the perspective of demand and supply and argued that demand is the main driving force. Chien [93] analyzed the correlation and fit between the geographical distribution of healthcare services and health needs and suggested a demand-oriented approach to the spatial allocation of public hospitals based on demand in Malaysia. Different from them, this study finds that there is some demand and supply mismatch for hospitals, doctors, and beds in China, and that the latter is more severe than the former. Besides, previous studies on geographic disparities in health care services have been limited by not considering the supply capacity of health care providers [94]. Using the supply-demand relationship as an analytical framework, this paper considers the provincial population demand, as well as the carrying capacity of the level of economic development, on the supply of medical resources, and it analyzes the types of supply and demand mismatches and their spatial effects, more in line with the actuality [95].

There is still much controversy regarding whether the spatial heterogeneity of medical resources is reasonable. This paper, in line with Shinjo [96] and Paramita [97], finds that interregional differences in medical resources in China, Japan, and Indonesia are small, and the Gini indices of hospitals, doctors, and beds are generally less than 0.4, indicating the achievement of spatially balanced development. In contrast, other scholars found that the intercontinental distribution of health resources is extremely unequal in Ethiopia, Mongolia, Iran, and Sudan [98]. For example, Woldemichael [99], Chavehpour [100], and Rezaei [101] found that the Gini index of medical resources in Ethiopia and Iran is generally greater than 0.4, and even up to 0.75; further, Erdenee [102] found that the Gini indices of doctors and beds in Mongolia were 0.74 and 0.69, respectively. In terms of trends, this paper finds that the Gini index of the geographical distribution of medical resources in China remains stable over time, and the spatial pattern becomes stabilized. In contrast, Costa [103] and Russo [104] argued that the inequality in the geographical distribution of hospitals and doctors is decreasing in Portugal and Brazil, and Horev [105] found a further increase in the Gini index in the United States, suggesting that the spatial distribution of health resources is becoming more uneven and such differences may be clearly related to the stage of development, national conditions, and health resource allocation criteria and methods in different countries [106]. The geographical distribution of health resources and their spatio-temporal evolution are uneven in many countries around the world, and it is necessary to consider critical values when promoting the optimization of the spatial layout of health resources, while taking into account spatial justice and allocation efficiency [107].

The temporal change process and spatial distribution pattern analysis of health resources are integrated based on Boston Consulting Group matrix, and the evolution trend is divided into four types, which helps develop differentiated and adaptive management policies for different provinces and identify the best combination of health resources in allocation. Provinces in dynamic matching should adopt a stable development strategy and maintain the same trend regardless of the type of evolution. Provinces in a positive mismatch should adopt an incremental expansion strategy, and the evolution trend of the question or star type should remain unchanged; otherwise, it should be changed from dog/cow to question/star. Provinces in the state of negative mismatch should adopt a smart shrink strategy, and the evolution trend of dog- or cow-type should remain unchanged; otherwise, it should be changed from question/star to dog/cow. For example, in the hospital dimension, Inner Mongolia, Fujian, Qinghai, and Ningxia have long been in dynamic matching, and they should adhere to the stable development strategy to maintain the evolution trend of the dog type in the future. Similarly, stable development strategies are adopted in Anhui, Hainan, Chongqing, Yunnan, Guizhou, Xinjiang (maintaining the question trend), Hubei, Guangxi (maintaining the cow trend), and Shandong (maintaining the star trend). Hebei, Hubei, Sichuan, and Shanxi have long been in positive mismatch, and they should adopt an expansion strategy to push the evolution trend from cow to star. Shanghai, Zhejiang, Jiangsu, and Guangdong have been in negative mismatch for a long time, and they should adopt a smart shrink strategy to reduce the growth rate and push the evolution trend from question to dog and from star to cow. In terms of doctors and beds, Beijing and Tianjin are in dynamic matching, and they should introduce a stable development strategy to maintain the dog evolution trend. Heilongjiang, Sichuan, Guizhou, and Yunnan have long been in positive mismatch, and they should adopt a smart shrink strategy in the future to maintain the evolution trend of dog or cow type, or to change from question to dog.

The comparison of spatio-temporal evolution trends and spatial mismatch relationships reveals the rationality of health resource supply and allocation schemes in the provinces and their improvement directions. For example, Zhejiang attaches great importance to investment in medical resources and, in recent years, has implemented the “13th Five-Year Plan” for the development of health care and plan for the medical service system in the province, which has contributed to the allocation of health resources in Zhejiang as a growth pole in China (in the evolution trend of star type). However, the spatial mismatch shifted from dynamic matching to negative mismatch from 2010 to 2020, indicating a shift from balance to imbalance between the large supply of health resources and population demand, with supply outstripping demand and extensive use of health facilities and resources. For example, the slowdown in investment in doctors and beds in Hebei in recent years has led to their evolution trend of cow-type. Notably, the type of spatial mismatch changed from dynamic matching in 2010 to positive mismatch in 2020, indicating the problem of undersupply of health facilities and resources, with the shift of supply-demand equilibrium of health services to disequilibrium, as a result of the long-term low investment in health care resources. In addition, despite the different evolution trends of health resources, such as question in Beijing and Tianjin, cow in Liaoning and Jiangxi, and dog in Inner Mongolia and Fujian, they all remained long in supply-demand balance of hospital allocation (in dynamic matching in 2010 and 2020).

In addition, it is an innovation to include spatial effects in the analysis of influence factors and measure their interaction effects in this paper. Unlike the published articles, this paper incorporates spatial heterogeneity and autocorrelation into the influence factor analysis, based on the geographic detector method, to measure the force of influence factors on the geographical distribution of medical resources and reveal the interaction effects of different influence factors, thus further improving the accuracy of the driving mechanism analysis. This paper finds that factors such as children and elderly population, health care government investment, service industry added value, high quality population, medical insurance fund expenditure, and government revenue have a great direct influence on the geographical distribution of health resources, which further validates the findings of some scholars. For example, Li [108], Zheng [109], and Guo [110] found that economic development, urbanization wage, population ageing, financial health expenditure levels, and population size are key factors affecting the geographical distribution of medical resources in China. Song [111], Ding [112], and Guo [113] found that social, economic, and environmental factors have great influence on the geographical clustering and spatio-temporal evolution trends of medical resources by leveraging the Bayesian local spatiotemporal regression model and spatial econometric model. In this paper, per capita GDP, urbanization rate and residents’ medical services consumption are found to be statistically insignificant in their direct influence, but their interaction influence, when combined with other factors, cannot be ignored. This is different from the analysis results of Yang [114] and Qian [115], who argued that these factors promote the geographic concentration of medical resources and are positively correlated with their spatio-temporal evolution. This difference may be caused by the different research methods. They adopted a statistical approach that did not take spatial effects into account, with the results emphasizing the consistency of “quantitative relationships”, rather than the similarity of “geographical relationships”.

### 5.2. Sustainable Development Spatial Strategies

**Figure 15:**
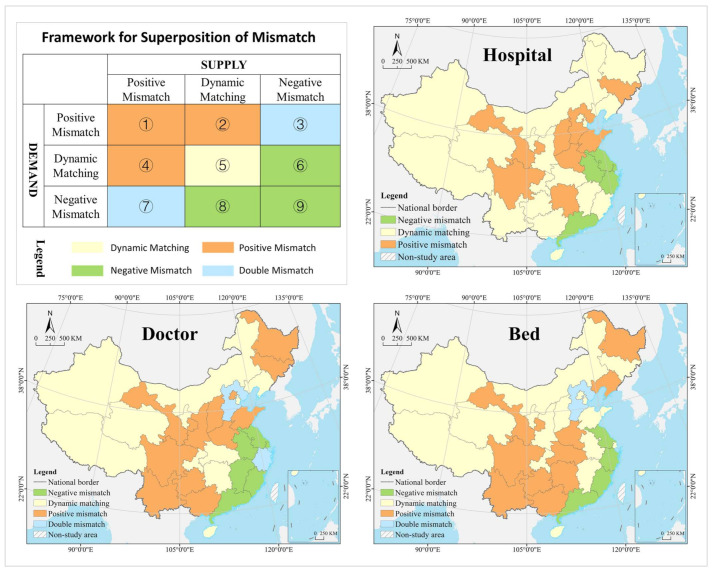
Spatial policy zoning for differentiated management.

To improve the uneven geographical distribution and spatial mismatch of health resources is one of the major challenges facing the government health sector. The government should redesign the spatial allocation scheme of medical resources and improve the supply and demand of health services, based on regional heterogeneity and spatial mismatch, under the guidance of spatial justice and regional health planning theories [116,117]. The first step is to advance the superposition analysis of the supply and demand mismatch quantitation results and divide the 16 combinations into 4 types, where (5) is of dynamic matching, indicating that medical resources have achieved a dynamic balance between supply and demand and are in an optimal state, and a path-dependent strategy should be adopted in the future policy design to maintain the current development trend and provide a reference for policy design in other types of regions [118]. Areas (1), (2), and (4) are of positive mismatch, indicating that the current medical resources are in excess of supply, beyond the actual demand and economic carrying capacity. There may have been a serious outflow of population in such areas, and a smart contraction strategy should be adopted in the future policy design to moderately control the supply of new medical resources. Areas (6), (8), and (9) are of negative mismatch, indicating that the supply of medical resources is less than the actual demand and lower than the carrying capacity of the economy. These areas are likely to be economically developed and attract a large foreign population. They should adopt smart growth strategies to increase the supply of and investment in new health resources in the future. Areas (3) and (7) are of double mismatch, indicating that the current medical resources fail to meet the actual demand, but are beyond the economic carrying capacity, so external support should be introduced in future policy design, or the current medical resources are already excessive and far below the economic carrying capacity, so new investment should be controlled with consideration of increasing external medical aid or cross-regional supportive transfer payments in the future policy design. The second step is that, in order to reduce the uncertainty of the spatial allocation of medical resources, the government should design differentiated management policies by spatial zoning, based on the four types generated by the superposition analysis [119,120] (Figure 15).

In the hospital dimension, Beijing, Tianjin, Fujian, Jiangxi, Hubei, Chongqing, Guizhou, Guangxi, Hainan, Yunnan, Tibet, Qinghai, Xinjiang, Ningxia, Shaanxi, Inner Mongolia, Liaoning, and Heilongjiang are of dynamic matching. Hebei, Shandong, Shanxi, Henan, Hunan, Sichuan, Gansu, and Jilin are of positive mismatch, and Guangdong, Shanghai, Jiangsu, Zhejiang, and Anhui are of negative mismatch. According to the development trend, the future policy design should follow the guidance as below: Beijing, Tianjin, Chongqing, Guizhou, Yunnan, Tibet, Xinjiang, Hainan, Guangdong, Jiangsu, and Zhejiang should continue to maintain high growth and focus on cultivating provinces with high potential by carefully analyzing the development drivers to promote them as regional medical service centers. Fujian, Qinghai, Ningxia, Inner Mongolia, Heilongjiang, Hebei, Shanxi, Henan, Hunan, and Sichuan should maintain their slow growth or reduction in quantity. Shandong, Gansu, and Jilin should implement contractionary policies for strict control of the amount of growth and reverse the development trend of high growth, in order to avoid the waste or risk brought about by blind investment. Shanghai, Anhui, Jiangxi, Hubei, Guangxi, Shaanxi, and Liaoning should introduce innovation policies and invest their limited resources primarily in new demand creation or new supply development.

In the doctor dimension, Tianjin, Hubei, Hunan, Chongqing, Tibet, Qinghai, Xinjiang, Ningxia, Inner Mongolia, and Liaoning are of dynamic matching. Beijing, Shandong, Shanxi, Henan, Shaanxi, Gansu, Sichuan, Yunnan, Guizhou, Guangxi, Liaoning, and Jilin are of positive mismatch. Shanghai, Jiangsu, Anhui, Fujian, Jiangxi, and Guangdong are of negative mismatch. Hebei and Zhejiang are of double mismatch. According to the development trend, the future policy design should follow the guidance as below: Jiangsu, Fujian, and Guangdong should continue to maintain high growth and cultivate them into regional integrated medical service centers. Shanghai, Anhui, Jiangxi, and Shandong should increase investment to further boost growth. Tianjin, Hubei, Hunan, Xinjiang, Inner Mongolia, Liaoning, Beijing, Shandong, Shanxi, Henan, Gansu, Liaoning, and Jilin should maintain a slow growth or reduction in quantity, while Tibet, Qinghai, Ningxia, Shaanxi, Sichuan, Yunnan, Guizhou, and Guangxi should introduce contractionary policies to strictly control the amount of growth and avoid waste or risk from blind investment. The current medical resources in Hebei fail to meet the actual demand and are beyond the economic carrying capacity, so external support is required in the future policy design. The current medical resources in Zhejiang are already excessive and far below the economic carrying capacity, so it should control the new investment and consider increasing external medical aid or cross-regional supportive transfer payments in the future policy design.

In the bed dimension, Beijing, Tianjin, Shandong, Anhui, Shanghai, Jiangxi, Hainan, Chongqing, Shanxi, Shaanxi, Ningxia, Qinghai, Tibet, Xinjiang, Inner Mongolia, and Jilin are of dynamic matching. Liaoning, Heilongjiang, Henan, Hubei, Hunan, Guangxi, Guizhou, Yunnan, Sichuan, and Gansu are of positive mismatch. Shandong, Fujian, Zhejiang, and Jiangsu are of negative mismatch. Hebei is of double mismatch. In light of the development trend, the future policy design should follow the guidance as below: Anhui, Jiangxi, Hainan, Chongqing, Shaanxi, Qinghai, Tibet, Fujian, Zhejiang, and Jiangsu should continue to maintain high growth. Shandong should increase investment to further boost its growth rate. Beijing, Tianjin, Shandong, Shanghai, Shanxi, Ningxia, Xinjiang, Inner Mongolia, Jilin, Liaoning, and Heilongjiang should maintain a slow growth or reduction in quantity. Henan, Hubei, Hunan, Guangxi, Guizhou, Yunnan, Sichuan, and Gansu should implement contractionary policies and strict control of the amount of growth, in order to avoid waste or risk brought about by blind investment. The current medical resources in Hebei fail to meet the actual demand and are beyond the economic carrying capacity, so external support is required in the future policy design.

In general, differentiated and adaptive management strategies should be adopted, depending on the relationship between health resource supply and demand, in order to maintain sustainable evolution trends or reverse unsound ones. For example, Hainan, Chongqing, Tibet, Qinghai, Ningxia, and Xinjiang, in dynamic matching, should implement a stable development strategy, with no need to change their current health resource allocation policy (which has already achieved a balance between supply and demand) and evolution trend. Sichuan and Henan, in positive mismatch, should implement an incremental expansion strategy. That is, they need to expand future investments in hospitals, doctors, and beds and drive the evolution trend to maintain or shift to question or star to address the problem of insufficient supply and to improve their health services and support capacity. Guangdong and Jiangsu, in negative mismatch, should implement a smart shrink strategy. In other words, it is necessary for them to reduce the future investment in health resources and promote the evolution trend to maintain or transform into dog or cow, in order to address the problem of oversupply and improve the efficiency of resource utilization. Hebei, Shanxi, Jilin, Heilongjiang, and Gansu, in double mismatch, should adopt an integrated control strategy. That is, due to the imbalance between the supply of health resources and population demand and the mismatch with economic carrying capacity, they should apply to the central government for inter-provincial transfer payments or medical assistance in the future to further improve their regional service capacity of health facilities and resources.

## 6. Conclusions

Reducing the inequality of geographical distribution and spatial mismatch of medical resources is still a key policy goal of health authorities in most countries [121]. Based on a combination of methods, such as Boston Consulting Group matrix, spatial mismatch index, spatial econometric model, and Geodetector, this paper analyzes the characteristics of geographic distribution of medical resources at provincial level in China and their driving mechanisms and spatio-temporal evolution trends, as well as the degree and types of spatial mismatch. It also proposes a differentiated management policy design, based on spatial zoning, which is of great value to promote the evidence-based decision making of medical resources spatial allocation scheme and health planning.

This paper finds that the geographical pattern of medical resources in China is solid over time, and that the quantity of resources and its change amplitude and speed have high spatial agglomeration and differentiation. The spatio-temporal evolution of inter-provincial medical resources in China has become diversified, with similar regions having high geographic agglomeration and weak spatial autocorrelation, as well as a gradient distribution of cold hot spots in a core-periphery structure. Children and elderly population, health care government investment, and service industry added value are the key factors influencing the geographical distribution of health resources, while social consumption, government revenue, high quality population, and medical insurance fund expenditure are important factors. The interaction between the different influence factors is dominated by bifactor enhancement, and about 30–40% of the factor pairs are in nonlinear enhancement. Although per capita GDP, urbanization rate, and residents’ medical services consumption do not have statistically significant direct influence, they have high interaction influence with other factors as auxiliary factors that cannot be ignored. Most of the provinces’ medical resources are in dynamic matching, mainly concentrated in west and north China and the middle reaches of the Yangtze River. A path-dependent strategy should be adopted in the future policy design to maintain the current development. The mismatch in the supply of medical resources in China is more serious than that in demand, and the proportion of mismatch in hospitals and beds is decreasing; however, the opposite is true for doctors. For the regions of positive mismatch, hospital demand mismatches and hospital, doctor, and bed supply mismatches are clustered, while doctor and bed demand mismatches are randomly distributed, so smart contraction-type strategies should be adopted in the future policy design to moderately control the supply of new medical resources and avoid the waste of resources or investment risks. For the regions of negative mismatch, doctor and bed demand mismatches, as well as doctor, hospital, and bed supply mismatches, are clustered in bands, while hospital demand mismatches are randomly distributed, so smart growth-oriented strategies should be adopted in the future to increase the supply and investment in new medical resources to achieve sustainable development. The hot and cold spots of supply and demand mismatch are highly clustered, shaping a spatial structure of center-periphery or gradient change. Hospital demand mismatches and hospital, doctor, and bed demand mismatches all have positive spatial autocorrelation and are increasingly spatially correlated and dependent; however, doctor and bed demand mismatches have changed from positive spatial autocorrelation to uncorrelation.

This paper is innovative in two areas. First, it introduces the spatial mismatch index to quantitatively measure the degree and type of medical resource mismatch at the provincial level in China from both the supply and demand perspectives and proposes a method for designing differential management policies, based on spatial zoning, which provides a basis for evidence-based decision making in health planning. Scholars are currently focusing on the study of mismatch of mobile factor resources, such as labor, capital, and commodities, and the immobility of health resources makes it more difficult and costly to correct their mismatch problems. In developing countries with limited health resources, incorporating economic carrying capacity and population demand power into health resource allocation models and health economics [122] and designing future health resource allocation policies, based on the types of mismatches, in line with the evolution trend, may help improve the equity and sustainability of the regional healthcare service system. Second, this paper incorporates spatial effects into the analysis of the driving mechanism of geographical distribution of medical resources, which deepens the research from the level of “quantitative relationship” consistency to the “geographical relationship” similarity and measures the interaction effects and relationships of different influence factors, thus significantly improving the precision of the analysis results.

In addition, this study is applicable to China; it also has great reference value for developing countries, such as Iran, Egypt, Ethiopia, South Africa, Tanzania, India, Malaysia, and Mongolia. As described above, they have very limited medical resources, with a striking problem of uneven geographical distribution and spatial mismatch. An empirical study of these countries, using the research framework and methodology provided in this paper, can help them design differentiated and adaptive health resource management policies. It should be noted that interest games and institution settings play an important role in the allocation of medical resources [123], but they are limited by data availability and are not included in this discussion, which is the shortcoming of this paper. In China, the supply of health resources is dominated by the government and institutionalized and hierarchical control of health resource allocation among different levels of government has led to intense competition between provinces, cities, and towns. Data on gaming and institutionalization of different participants is part of tacit knowledge. It is not included in official statistics; to acquire such information is also difficult through unofficial channels. The data shortage leads to less comprehensive analysis results, and it may compromise the accuracy of the conclusions, to some extent, which is the direction of future research efforts. In addition, the spatial mismatch index is essentially a static model, but the real world is dynamic, so it is necessary to identify or create new methods that can dynamically analyze health resource mismatch in the future to further improve the credibility and applicability of the analysis results.

## Figures and Tables

**Table 1:** Variables and data sources.

Type	Code	Indicator	Data Source
Dependent variable	$Y_{1}$	Hospital	*China statistical yearbook* *China health statistical yearbook*
	$Y_{2}$	Doctor	
	$Y_{3}$	Bed	
Independent variable	$X_{1}$	Service industry added value	*China statistical yearbook* *Provincial statistical yearbook*
	$X_{2}$	Per capita GDP	
	$X_{3}$	Government revenue	
	$X_{4}$	Social consumption	
	$X_{5}$	Urbanization rate	
	$X_{6}$	Children and elderly population	
	$X_{7}$	High quality (university or above) population	
	$X_{8}$	Health care government investment	
	$X_{9}$	Residents’ medical services consumption	
	$X_{10}$	Medical insurance fund expenditure	
Demand and supply variable	$Z_{1}$	Population	
	$Z_{1}$	GDP	

**Table 2:** Parameters for global autocorrelation analysis.

Indicator Name			Global Moran’s I	*p*	Z
Evolution trend	Hospitals		0.14	0.10	1.39
	doctors		0.15	0.08	1.45
	beds		0.23	0.02	2.23
Spatial mismatch type of population	Hospitals	2010	0.21	0.03	2.06
		2020	0.28	0.00	2.69
	Doctors	2010	0.15	0.06	1.67
		2020	−0.10	0.30	−0.55
	Beds	2010	0.18	0.05	1.72
		2020	0.07	0.21	0.80
Spatial mismatch type of GDP	Hospitals	2010	0.23	0.03	2.24
		2020	0.24	0.02	2.42
	Doctors	2010	0.30	0.01	2.81
		2020	0.46	0.00	4.25
	Beds	2010	0.15	0.07	1.49
		2020	0.19	0.04	1.94

**Table 3:** Analysis on influence factors of medical resources changes.

**Indicator**		$X_{1}$	$X_{2}$	$X_{3}$	$X_{4}$	$X_{5}$	$X_{6}$	$X_{7}$	$X_{8}$	$X_{9}$	$X_{10}$	$\bar{X}$
Hospital	q	0.54	0.03	0.48	0.33	0.06	0.80	0.34	0.58	0.10	0.36	0.52
	*P*	0.01	0.34	0.05	0.08	0.17	0.00	0.04	0.02	0.09	0.04	0.05
Doctor	q	0.74	0.03	0.66	0.83	0.00	0.92	0.73	0.87	0.04	0.76	0.79
	*P*	0.00	0.57	0.01	0.00	0.92	0.00	0.00	0.00	0.29	0.00	0.05
Bed	q	0.71	0.08	0.59	0.75	0.01	0.95	0.60	0.83	0.07	0.64	0.72
	*P*	0.00	0.66	0.02	0.00	0.65	0.00	0.01	0.00	0.16	0.00	0.05

Note: $\bar{X}$ represents the average value of $X_{1}$~$X_{10}$, *p* < 0.10.

**Table 4:** Factor interaction analysis of the hospital.

	$X_{1}$	$X_{2}$	$X_{3}$	$X_{4}$	$X_{5}$	$X_{6}$	$X_{7}$	$X_{8}$	$X_{9}$	$X_{10}$
$X_{1}$	0.54									
$X_{2}$	0.69	0.03								
$X_{3}$	0.72	0.73	0.48							
$X_{4}$	0.63	0.61	0.74	0.33						
$X_{5}$	0.72	0.09	0.73	0.64	0.06					
$X_{6}$	0.85	0.88	0.88	0.91	0.87	0.80				
$X_{7}$	0.59	0.64	0.60	0.48	0.64	0.84	0.34			
$X_{8}$	0.73	0.69	0.76	0.64	0.69	0.87	0.67	0.58		
$X_{9}$	0.64	0.16	0.67	0.51	0.14	0.83	0.57	0.63	0.10	
$X_{10}$	0.66	0.69	0.61	0.45	0.71	0.85	0.47	0.72	0.63	0.36

**Table 5:** Factor interaction analysis of the doctor.

	$X_{1}$	$X_{2}$	$X_{3}$	$X_{4}$	$X_{5}$	$X_{6}$	$X_{7}$	$X_{8}$	$X_{9}$	$X_{10}$
$X_{1}$	0.74									
$X_{2}$	0.81	0.03								
$X_{3}$	0.81	0.81	0.66							
$X_{4}$	0.91	0.89	0.92	0.83						
$X_{5}$	0.85	0.07	0.83	0.91	0.00					
$X_{6}$	0.97	0.96	0.97	0.98	0.96	0.92				
$X_{7}$	0.83	0.84	0.88	0.92	0.85	0.97	0.73			
$X_{8}$	0.95	0.89	0.93	0.93	0.89	0.97	0.96	0.87		
$X_{9}$	0.83	0.21	0.81	0.85	0.10	0.93	0.87	0.88	0.04	
$X_{10}$	0.93	0.92	0.90	0.93	0.93	0.98	0.80	0.94	0.91	0.76

**Table 6:** Factor interaction analysis of the bed.

	$X_{1}$	$X_{2}$	$X_{3}$	$X_{4}$	$X_{5}$	$X_{6}$	$X_{7}$	$X_{8}$	$X_{9}$	$X_{10}$
$X_{1}$	0.71									
$X_{2}$	0.88	0.08								
$X_{3}$	0.78	0.83	0.59							
$X_{4}$	0.87	0.95	0.89	0.75						
$X_{5}$	0.85	0.17	0.79	0.91	0.01					
$X_{6}$	0.97	0.99	0.97	0.98	0.97	0.95				
$X_{7}$	0.81	0.90	0.86	0.86	0.83	0.97	0.60			
$X_{8}$	0.93	0.92	0.90	0.94	0.89	0.98	0.91	0.83		
$X_{9}$	0.79	0.27	0.74	0.80	0.10	0.96	0.81	0.87	0.07	
$X_{10}$	0.90	0.90	0.87	0.91	0.90	0.97	0.68	0.93	0.89	0.64

## Data Availability

https://data.cnki.net/Yearbook/Single/N2022010155 (accessed on 15 May 2022) and http://www.stats.gov.cn/tjsj/ndsj/ (accessed on 11 May 2022).

## Appendix A

**Table A1:** Dependent, demand, and supply variable data, based on sum standardization method.

City	Hospitals		Doctors		Beds		GDP		Population	
	2010	2020	2010	2020	2010	2020	2010	2020	2010	2020
Beijing	0.0100	0.0104	0.0273	0.0258	0.0194	0.0140	0.0323	0.0357	0.0147	0.0155
Tianjin	0.0048	0.0057	0.0118	0.0106	0.0102	0.0075	0.0211	0.0139	0.0097	0.0098
Hebei	0.0869	0.0850	0.0534	0.0501	0.0522	0.0486	0.0467	0.0358	0.0539	0.0529
Shanxi	0.0439	0.0402	0.0337	0.0261	0.0326	0.0246	0.0211	0.0174	0.0268	0.0248
Inner Mongolia	0.0241	0.0240	0.0206	0.0189	0.0195	0.0178	0.0267	0.0171	0.0185	0.0170
Liaoning	0.0371	0.0334	0.0386	0.0298	0.0427	0.0346	0.0422	0.0248	0.0328	0.0302
Jilin	0.0207	0.0250	0.0228	0.0203	0.0240	0.0190	0.0198	0.0122	0.0206	0.0170
Heilongjiang	0.0236	0.0200	0.0320	0.0231	0.0334	0.0278	0.0237	0.0135	0.0287	0.0225
Shanghai	0.0050	0.0058	0.0210	0.0194	0.0220	0.0167	0.0393	0.0382	0.0173	0.0176
Jiangsu	0.0330	0.0349	0.0560	0.0611	0.0563	0.0588	0.0948	0.1015	0.0590	0.0601
Zhejiang	0.0320	0.0336	0.0430	0.0490	0.0385	0.0397	0.0634	0.0638	0.0408	0.0459
Anhui	0.0245	0.0287	0.0377	0.0374	0.0393	0.0448	0.0283	0.0382	0.0447	0.0433
Fujian	0.0288	0.0275	0.0243	0.0261	0.0236	0.0238	0.0337	0.0434	0.0277	0.0295
Jiangxi	0.0364	0.0359	0.0282	0.0273	0.0260	0.0314	0.0216	0.0254	0.0335	0.0320
Shandong	0.0715	0.0830	0.0788	0.0763	0.0799	0.0711	0.0896	0.0722	0.0719	0.0721
Henan	0.0808	0.0730	0.0721	0.0698	0.0684	0.0733	0.0528	0.0543	0.0705	0.0705
Hubei	0.0366	0.0347	0.0426	0.0400	0.0419	0.0452	0.0365	0.0429	0.0429	0.0407
Hunan	0.0634	0.0548	0.0452	0.0456	0.0488	0.0571	0.0367	0.0413	0.0493	0.0471
Guangdong	0.0479	0.0546	0.0723	0.0747	0.0627	0.0621	0.1053	0.1094	0.0783	0.0895
Guangxi	0.0349	0.0331	0.0325	0.0351	0.0300	0.0325	0.0219	0.0219	0.0346	0.0356
Hainan	0.0050	0.0060	0.0063	0.0070	0.0054	0.0064	0.0047	0.0055	0.0065	0.0072
Chongqing	0.0187	0.0205	0.0195	0.0224	0.0216	0.0259	0.0181	0.0247	0.0216	0.0228
Sichuan	0.0793	0.0809	0.0570	0.0612	0.0629	0.0714	0.0393	0.0480	0.0603	0.0594
Guizhou	0.0271	0.0282	0.0188	0.0272	0.0220	0.0304	0.0105	0.0176	0.0261	0.0274
Yunnan	0.0244	0.0260	0.0253	0.0341	0.0328	0.0357	0.0165	0.0242	0.0345	0.0335
Tibet	0.0053	0.0068	0.0020	0.0030	0.0018	0.0020	0.0012	0.0019	0.0023	0.0026
Shaanxi	0.0381	0.0342	0.0317	0.0331	0.0297	0.0299	0.0232	0.0259	0.0280	0.0280
Gansu	0.0285	0.0256	0.0168	0.0170	0.0189	0.0189	0.0094	0.0089	0.0192	0.0177
Qinghai	0.0062	0.0063	0.0043	0.0048	0.0043	0.0045	0.0031	0.0030	0.0042	0.0042
Ningxia	0.0044	0.0045	0.0048	0.0053	0.0049	0.0045	0.0039	0.0039	0.0047	0.0051
Xinjiang	0.0171	0.0178	0.0194	0.0182	0.0243	0.0199	0.0124	0.0136	0.0164	0.0184

**Table A2:** Independent variable data based on sum standardization method in 2020.

City	$X_{1}$	$X_{2}$	$X_{3}$	$X_{4}$	$X_{5}$	$X_{6}$	$X_{7}$	$X_{8}$	$X_{9}$	$X_{10}$
Beijing	0.0550	0.0751	0.0548	0.0350	0.0443	0.0124	0.0597	0.0321	0.0591	0.0593
Tianjin	0.0165	0.0463	0.0192	0.0091	0.0429	0.0088	0.0211	0.0093	0.0446	0.0162
Hebei	0.0340	0.0221	0.0382	0.0324	0.0304	0.0574	0.0381	0.0433	0.0292	0.0413
Shanxi	0.0164	0.0230	0.0229	0.0172	0.0317	0.0230	0.0267	0.0230	0.0328	0.0208
Inner Mongolia	0.0154	0.0328	0.0205	0.0121	0.0342	0.0147	0.0200	0.0199	0.0321	0.0147
Liaoning	0.0244	0.0268	0.0265	0.0229	0.0365	0.0274	0.0380	0.0219	0.0394	0.0298
Jilin	0.0117	0.0231	0.0108	0.0098	0.0317	0.0148	0.0211	0.0159	0.0349	0.0130
Heilongjiang	0.0123	0.0194	0.0115	0.0130	0.0332	0.0186	0.0227	0.0213	0.0348	0.0197
Shanghai	0.0515	0.0710	0.0704	0.0407	0.0452	0.0146	0.0510	0.0289	0.0506	0.0495
Jiangsu	0.0981	0.0552	0.0905	0.0946	0.0372	0.0599	0.0727	0.0534	0.0343	0.0754
Zhejiang	0.0655	0.0459	0.0724	0.0680	0.0365	0.0388	0.0524	0.0444	0.0333	0.0639
Anhui	0.0360	0.0289	0.0321	0.0468	0.0295	0.0471	0.0352	0.0404	0.0262	0.0350
Fujian	0.0379	0.0482	0.0307	0.0475	0.0348	0.0285	0.0284	0.0277	0.0271	0.0264
Jiangxi	0.0225	0.0259	0.0250	0.0265	0.0306	0.0344	0.0223	0.0340	0.0250	0.0264
Shandong	0.0712	0.0329	0.0655	0.0746	0.0319	0.0775	0.0644	0.0554	0.0330	0.0693
Henan	0.0487	0.0253	0.0416	0.0574	0.0281	0.0820	0.0464	0.0575	0.0279	0.0526
Hubei	0.0405	0.0339	0.0251	0.0459	0.0318	0.0402	0.0406	0.0540	0.0299	0.0371
Hunan	0.0393	0.0287	0.0300	0.0415	0.0297	0.0514	0.0325	0.0391	0.0349	0.0380
Guangdong	0.1137	0.0402	0.1291	0.1026	0.0375	0.0778	0.0854	0.0939	0.0283	0.0889
Guangxi	0.0209	0.0202	0.0171	0.0200	0.0274	0.0404	0.0227	0.0331	0.0267	0.0289
Hainan	0.0061	0.0251	0.0081	0.0050	0.0305	0.0069	0.0064	0.0117	0.0240	0.0062
Chongqing	0.0240	0.0356	0.0209	0.0301	0.0352	0.0238	0.0222	0.0230	0.0364	0.0223
Sichuan	0.0463	0.0265	0.0425	0.0531	0.0287	0.0622	0.0482	0.0546	0.0328	0.0527
Guizhou	0.0165	0.0211	0.0178	0.0200	0.0269	0.0309	0.0198	0.0300	0.0227	0.0205
Yunnan	0.0230	0.0237	0.0211	0.0250	0.0253	0.0322	0.0248	0.0377	0.0276	0.0272
Tibet	0.0017	0.0239	0.0022	0.0019	0.0181	0.0025	0.0020	0.0076	0.0109	0.0016
Shaanxi	0.0228	0.0302	0.0225	0.0245	0.0317	0.0273	0.0330	0.0270	0.0367	0.0223
Gansu	0.0090	0.0164	0.0087	0.0093	0.0264	0.0180	0.0162	0.0196	0.0274	0.0135
Qinghai	0.0028	0.0232	0.0030	0.0022	0.0304	0.0039	0.0040	0.0091	0.0349	0.0046
Ningxia	0.0036	0.0248	0.0042	0.0033	0.0329	0.0049	0.0058	0.0063	0.0333	0.0047
Xinjiang	0.0129	0.0244	0.0148	0.0078	0.0286	0.0176	0.0166	0.0250	0.0292	0.0182
